# A general Fc engineering platform for the next generation of antibody therapeutics

**DOI:** 10.7150/thno.51299

**Published:** 2021-01-01

**Authors:** Da Chen, Yingjie Zhao, Mingyu Li, Hang Shang, Na Li, Fan Li, Wei Wang, Yuan Wang, Ruina Jin, Shiyu Liu, Xun Li, Shan Gao, Yujie Tian, Ruonan Li, Huanhuan Li, Yongyan Zhang, Mingjuan Du, Youjia Cao, Yan Zhang, Xin Li, Yi Huang, Liaoyuan A. Hu, Fubin Li, Hongkai Zhang

**Affiliations:** 1 State Key Laboratory of Medicinal Chemical Biology and College of Life Sciences, Nankai University, 94 Weijin Road, Tianjin, 300071, China.; 2 Shanghai Institute of Immunology, Faculty of Basic Medicine, Shanghai Jiao Tong University School of Medicine, Shanghai 200025, China.; 3 Key Laboratory of Cell Differentiation and Apoptosis of Chinese Ministry of Education, Shanghai Jiao Tong University School of Medicine, Shanghai 200025, China.; 4 Shanghai Institute for Advanced Immunochemical Studies, ShanghaiTech University, Shanghai, 201210, China.; 5 Amgen Research, Amgen Biopharmaceutical R&D (Shanghai) Co., Ltd, Shanghai, 201210, China.; 6 Department of Analytical Science, Zhenge Biotech, Shanghai, 201318, China.

**Keywords:** antibody therapeutics, Fc engineering, Glycoengineering, mammalian cell display, Fc gamma receptors

## Abstract

**Rationale:** Fc engineering has become the focus of antibody drug development. The current mutagenesis and *in silico* protein design methods are confined by the limited throughput and high cost, while the high-throughput phage display and yeast display technologies are not suitable for screening glycosylated Fc variants. Here we developed a mammalian cell display-based Fc engineering platform.

**Methods:** By using mammalian cell display and next generation sequencing, we screened millions of Fc variants for optimized affinity and specificity for FcγRIIIa or FcγRIIb. The identified Fc variants with improved binding to FcγRIIIa were substituted into trastuzumab and rituximab and the effector function of antibodies were examined in the PBMC-based assay. On the other hand, the identified Fc variants with selectively enhanced FcγRIIb binding were applied to CD40 agonist antibody and the activities of the antibodies were measured on different cell assays. The immunostimulatory activity of CD40 antibodies was also evaluated by OVA-specific CD8^+^ T cell response model in FcγR/CD40-humanized mice.

**Results:** Using this approach, we screened millions of Fc variant and successfully identified several novel Fc variants with enhanced FcγRIIIa or FcγRIIb binding. These identified Fc variants displayed a dramatic increase in antibody-dependent cellular cytotoxicity in PBMC-based assay. Novel variants with selectively enhanced FcγRIIb binding were also identified. CD40 agonist antibodies substituted with these Fc variants displayed activity more potent than the parental antibody in the *in vitro* and* in vivo* models*.*

**Conclusions:** This approach increased the throughput of Fc variant screening from thousands to millions magnitude, enabled screening variants containing multiple mutations and could be integrated with glycoengineering technology, represents an ideal platform for Fc engineering. The initial efforts demonstrated the capability of the platform and the novel Fc variants could be substituted into nearly any antibody for the next generation of antibody therapeutics.

## Introduction

Antibody drugs, with the efficacy, safety and specificity, are among the most effective therapies nowadays. Most of antibody therapeutics are IgG isotype 1, 2. For many of them, the efficacy is dependent on the engagement of Fc (fragment crystallizing) of antibody with the Fc gamma receptors (FcγRs). According to their signal transduction pattern, human FcγRs can be classified into activating receptors including FcγRI, FcγRIIa (131H or 131R alleles), FcγRIIc, FcγRIIIa (158V or 158F alleles), inhibitory receptor FcγRIIb and FcγRIIIb whose actual function has remained uncertain 3.

The activity of an antibody can be modulated by engineering its IgG Fc affinity to FcγRs. For example, antibody-dependent cellular cytotoxicity (ADCC) is governed by the engagement of Fc region with FcγRIIIa on Natural Killer (NK) cells. Glycoengineering or mutagenesis increases the affinity of Fc for FcγRIIIa on immune effector cells, leading to enhanced ADCC 2, 4-11. For example, Shields *et al.* comprehensively mapped the binding site on IgG1 for human FcγRs and FcRn by alanine scanning of all solvent-exposed amino acids in CH2 and CH3 domains of IgG and identified mutations that improved binding to the receptors 5. A few groups reported that the fucose deficient IgG1 exhibited enhanced binding to human FcγRIIIa and antibody-dependent cellular cytotoxicity 4, 8. Several therapeutic antibodies with engineered Fc, such as obinutuzumab, mogamulizumab and recently inebilizumab are entering the clinic and demonstrating clinical benefit 12-14.

Besides modulation of the effector functions, crosslinking of Fc by FcγRIIb expressed on tumor infiltrating immune cells is considered essential for the antitumor activity of many agonistic antibodies targeting tumor necrosis factor receptor (TNFR) superfamily members in murine models 15. Fc domain of CD40 agonist antibody APX005M, which is now in phase 2 clinical trial, was engineered to increase binding to FcγRIIb based on the finding in a murine model that the efficacy of a CD40 agonist can be improved by increasing the Fc binding affinity to FcγRIIb 16.

There are several platforms to engineer the Fc fragment. Lazar *et al*. designed Fc variants with optimized FcγRIIIa affinity using protein design algorithms and experimentally screened thousands of designed variants 9. The successful design of Fc variant was impressive. Nevertheless, the computational method remains computationally expensive and is, for the time being, not applicable in a high throughput manner. Mimoto *et al*. purified and screened over 500 Fc variants with single substitution of amino acid, and identified several variants with enhanced affinity for FcγRIIb 17. Both methods are confined by the limited throughput and used an iterative approach where single mutations are identified separately and then combined.

To overcome these limitations, a high-throughput platform is imperative to screen for Fc variants containing multiple mutations. The multiple mutations could form epistatic interactions and they can't be identified by the two-step approach where single mutations are identified and then combined. The most widely used high-throughput protein engineering technology is the *in vitro* display technology, including phage display and yeast display 18, 19. However, phage-displayed proteins lacked glycosylation and glycoproteins displayed by Saccharomyces cerevisiae possessed high mannose glycan 20. Thus, they are not ideal platforms to screen Fc variants, given that the glycosylation of Fc region has a profound impact on its interaction with FcγRs. The above obstacles can be resolved by mammalian cell display technology, which could display the antibodies with correct folding and post translational modification on the surface of mammalian cells 21.

Here, we developed a mammalian cell display-based platform for screening of antibody Fc variants with improved binding property for FcγRs. Trastuzumab and rituximab with enhanced FcγRIIIa binding exhibited more effective ADCC effect and thus the capacity of the targeted antibody to trigger the death of cancerous cells. CD40 agonist antibodies with selectively enhanced FcγRIIb binding showed remarkable enhancement of immunostimulatory activity *in vitro* and *in vivo*. Therefore, the technology presented here allows for high-throughput screening of novel Fc variants with sought-after features which can be substituted into nearly any antibodies for the next generation therapeutic antibodies.

## Methods

### Cell culture

HEK293T cells were cultured in DMEM medium (Thermo Fisher Scientific). Jurkat-NFAT-FcγRIIIa reporter cell line, CD40 reporter cell line and Ramos cells were cultured in RPMI 1640 medium (Thermo Fisher Scientific). SK-BR-3 cells were cultured in McCoy's 5A (Modified) medium (Biological Industries). All the culture medium was supplemented with 10% fetal bovine serum (Biological Industries), 1× non-essential amino acids, 100 U/mL penicillin, 100 μg/mL streptomycin and 12.5 mM HEPES (Thermo Fisher Scientific). HEK293F cells were suspension cultured in FreeStyle™ 293 Expression Medium (Thermo Fisher Scientific). CHO-K1 or *FUT8*^-/-^ CHO-K1 cells were cultured in FreeStyle™ CHO Expression Medium (Thermo Fisher Scientific). All the cells were maintained in CO_2_ incubator at 37 °C.

### Mice

FcγR/CD40-humanized mice have been described previously and were kindly provided by Dr Jeffrey Ravetch (The Rockefeller University) 22. Mice were bred and maintained in the specific-pathogen-free environment at the Department of Laboratory of Animal Science, Shanghai Jiao Tong University School of Medicine. All animal care and study were performed in compliance with institutional and NIH guidelines and had been approved by SJTUSM Institutional Animal Care and Use Committee (Protocol Registry Number: A-2015-014).

### Protein preparation

The extracellular domains of human FcγRIIb, FcγRIIIa^F158^, FcγRIIIa^V158^, FcγRIIa^H131^ or FcγRIIa^R131^ with AviTag and a 6×his tag fused at the C-terminus were expressed by HEK293F cells and purified with HisTrap™ HP column (GE healthcare) and buffer-exchanged to PBS. For biotinylation, the purified FcγRs were treated with biotin-protein ligase (GeneCopoeia) and the degree of biotinylation was verified by a pull-down assay using streptavidin magnetic beads (Thermo Fisher Scientific). The Fc variant genes were obtained by site-directed mutagenesis using the wild-type Fc gene as template. Fc and antibody (rituximab, trastuzumab or NK003) variants were expressed by HEK293F or CHO-K1 cells and the defucosylated Fc antibodies were expressed by *FUT8*^-/-^ CHO-K1 cells and purified using protein A beads (GE healthcare).

### Fc library construction

The Fc library construction process was illustrated in Figure S1. The mammalian cell display vector is based on pCDH-CMV-MCS-EF1α-Puro lentivector (System Biosciences) and contains IL-2 signal peptide, FLAG-tag, GGGGS spacer, two in frame restriction sites (EcoRI and NheI), 218 linker (GSTSGSGKPGSGEGSTKG) and PDGFR transmembrane domain.

Fc libraries were constructed by overlap PCR. Fragment 1 was amplified by primer Lenti-F1 and a mixture of degenerate oligonucleotides. Fragment 2 was amplified by primers Lenti-F2 and Lenti-R2. The two fragments were assembled by overlap PCR and digested by EcoRI and NheI. The digested products were inserted into the mammalian cell display vector between the EcoRI and NheI restriction sites and transformed into chemically competent cells (TransGen Biotech).

### Mammalian cell display and cell sorting

Mammalian cell display vector harboring Fc libraries was transfected into HEK293T cells, together with pMDLg/pRRE, pRSV-Rev and pCMV-VSV-G packaging plasmids, using polyethylenimine (PEI), to produce lentivirus library. The lentivirus was concentrated with Lentivirus Concentration Reagent (BIOMIGA), and the titer was measured with Lenti-X™ p24 Rapid Titer Kit (Takara Bio Inc.).

1×10^7^ HEK293T cells were infected by lentivirus library at low MOI, followed by 48 h of culture to display Fc variants on the cell surface. The cells were then detached by StemPro Accutase Cell Dissociation Reagent (Thermo Fisher Scientific), resuspended in 1 mL blocking buffer (PBS supplemented with 5% FBS (V/V), 1% glucose (W/V), 1 mM Na_2_EDTA, 1 mM HEPES) and incubated for 30 min at 4 °C with gentle agitation. Then the cells were incubated with 250 nM (125 nM for the 2^nd^ and 3^rd^ rounds of sorting) biotinylated FcγRs (bio-FcγRs) in blocking buffer at 4 °C for 30 min. After washing three times with ice-cold FACS buffer (PBS supplemented with 1% glucose (W/V), 1 mM Na_2_EDTA, 1 mM HEPES), the cells were resuspended in 1 mL of blocking buffer containing streptavidin-PE (1:1000 dilution, Thermo Fishier Scientific) and ANTI-FLAG® M2-FITC (1:200 dilution, SIGMA) and incubated at 4 °C for 30 min. The cells were finally washed three times and resuspended in ice-cold FACS buffer for sorting (FACSAria III, BD).

### Next generation sequencing (NGS)

5×10^5^ cells before or after sorting were pelleted and lysed by 100 μL lysis buffer (91 μL deionized water, 5 μL 1M KCl, 1 μL 1M Tris-HCl (pH 9.0), 1 μL 10% Triton X-100 (Sigma), 2 μL proteinase K (NEB)). Cell lysate was used as template, the Fc variants coding region was PCR-amplified from the genome by primers F1 (sequence: 5′-GACAAAGGCGGTGGCGGTTCGAATTCGGAGCCCAAATCTTG-3′) and R1 (sequence: 5′-CTGGTGGAGCCGGATCCACCGCTAGCTTATCATTTACCCGGAGACAG-3′). Barcode for each library was added to the above PCR product through a second-round PCR, using the following primers for each library:

- Library1: F2: 5′- (barcode) GACAAAGGCGGTGGC-3′, R2: 5′- (barcode) CACGCCGTCCACGTAC-3′.
- Library2: F3: 5′- (barcode) AAAACTCACACATGC-3′; R3: 5′- (barcode) CAGCCAGTCCTGGTG-3′.
- Library3: F4: 5′- (barcode) CTCATGATCTCCCGG-3′; R4 5′- (barcode) TCGGGGCTGCCCTTTG-3′.
- Library4: F5: 5′- (barcode) GAGGTGCATAATGCC-3′; R5: 5′- (barcode) GCTGGGATAGAAGCC-3′.

200 ng of PCR products of each library were mixed and high-throughput sequenced using HiSeq X Ten System (Illumina).

### Data processing and analysis

The FASTQ file contained all the NGS data of the 12 samples (4 original libraries, 4 FcγRIIb-enriched libraries and 4 FcγRIIIa-enriched libraries), fastp was used to filter low-quality reads and remove adapters for the FASTQ file with default parameters. And specific barcodes for each sample were used to demultiplex the whole NGS date into individual FASTQ files in the software CLC Genomics Workbench v11. Then for each sample, the consistent regions located upstream and downstream of the mutation region were trimmed with maximum error rate 0.1 in CLC Genomics Workbench v11. After calculating the copy number and frequency of all type of variants at DNA level in each sample, individual variants were ranked by its copy number and then the cumulative curves were plotted for the before sorting and after sorting samples of library 1-4.

For each sample, the mutation regions were translated into amino acids sequences at reading frame +1 in CLC Genomics Workbench v11, and the copy number and frequency of each variants at amino acids level were calculated. Sequence logos of the 12 samples were generated by Weblogo3.6.02, for each variable amino acid position, the probability of each of the 20 naturally encoded amino acids was shown in Weblogo. The hydrophobic amino acids G, A, V, L, I, M, P, F, Y, W were colored in orange. The positively charged amino acids R, K, H were colored in red. The negatively charged amino acids D, E were colored in green. And the polar uncharged amino acids C, S, T, N, Q were colored in blue.

### Surface plasmon resonance (SPR) analysis

SPR experiments were performed using Biacore T200 SPR system (GE Healthcare) in HBS-EP+ buffer at 20 °C. Anti-his antibody was immobilized on a Series S CM5 chip through amine coupling. Then 500 nM of his-tagged soluble human FcγRs extracellular domains (FcγRIIb, FcγRIIIa^F158^, FcγRIIIa^V158^, FcγRIIa^H131^ and FcγRIIa^R131^ were produced in-house. FcγRI and FcRn were purchased from ACROBiosystems) were captured by the anti-his antibody in different flow cells. Fc variants samples were injected through the flow cells to analyze binding capacities.

To measure the equilibrium dissociation constant (K_D_), twofold serially diluted trastuzumab, rituximab or NK003 variants were injected through the flow cells for 120 s for association followed by a 130 s dissociation phase at a flow rate of 30 μL min^-1^. Prior to next assay cycle, the sensor chip surface was regenerated with Glycine-HCl (pH 1.5) for 30 s at a flow rate of 30 μL min^-1^. K_D_ values were calculated using the 1:1 binding kinetics model or steady state affinity model built in the Biacore T200 Evaluation Software.

### Antibody-dependent cellular cytotoxicity (ADCC) assay

Peripheral blood mononuclear cells (PBMCs) were isolated from the blood of healthy human donors by density gradient centrifugation (Ficoll-Paque™ Plus, GE Healthcare). ADCC was measured by the CytoTox 96® Non-Radioactive Cytotoxicity Assay (Promega Corporation). In brief, 1×10^4^ SK-BR-3 cells or Ramos cells (target cells) were incubated with ten-fold gradient diluted antibodies (for ADCC curve) or 1 μg/mL of trastuzumab (for three donors ADCC assay) in complete RPMI 1640 medium (without phenol red, Thermo Fisher Scientific) at 37 °C for 30 min, and then 1.5×10^5^ PBMCs (effector cells) were added with an effector/target ratio of 15:1. After 4 h of incubation, lactate dehydrogenase (LDH) activity in the supernatants was measured. Each measurement was performed with three repeats. The percentage of ADCC was calculated as follows: % Cytotoxicity = (experimental lysis - spontaneous effector lysis - spontaneous target lysis) / (maximum target lysis - spontaneous target lysis) × 100.

### FcγRIIIa reporter cell assay

96-well non-tissue culture treated plate wells were coated with 1 μg Fc variant protein per well overnight at 4 °C, and the wells were washed once with PBS and added with 1×10^5^ Jurkat-NFAT-FcγRIIIa reporter cell line. After 24 h of incubation in CO_2_ incubator at 37 °C, the luciferase activity of cells was detected using Luciferase Assay System (Promega Corporation).

### CD40 reporter cell assay

HEK293T cells were transfected with full length FcγRIIb, FcγRIIa^H131^ or FcγRIIa^R131^ expressing plasmids. After 24 h of expression, cells were dispersed by StemPro Accutase Cell Dissociation Reagent (Thermo Fisher Scientific) and seeded to 96-well plate with 5×10^4^ per well followed by 6 h of adherence. NK003 variants and 5×10^4^ CD40 reporter cells were resuspended in 200 μL of complete RPMI 1640 medium and added to the well. GFP expression in CD40 reporter cell was detected after 6 h or 24 h by flow cytometry (LSR Fortessa, BD) and mean fluorescence intensity (MFI) was calculated by FlowJo software.

### B cell activation

B cells were isolated from healthy donor PBMCs using CD19 MicroBeads (Miltenyi Biotec). 1×10^5^ B cells were seeded in 96-well plate with 100 μL complete RPMI medium, and ten-fold gradient diluted NK003 variants (100 μg/mL - 0.001 μg/mL) or IgG1 isotype control antibody (100 μg/mL) were added to each well. After 12 h (for CD23) or 48 h (for CD86 and HLA-DR) of stimulation, B cells were pelleted and blocked with PBS supplemented with 5% FBS and then stained with PE anti-human CD19 in combination with FITC anti-human CD23 or APC anti-human CD86 or Alexa Fluor 488 anti-human HLA-DR antibodies (BioLegend) at 4 ℃ for 30 min. B cells were gated as CD19^+^ and the expression level of the activation markers CD23, CD86 and HLA-DR were measured by flow cytometry (LSR Fortessa, BD). Mean fluorescence intensity (MFI) of FITC or APC or Alexa Fluor 488 was calculated using FlowJo software.

### OVA-specific CD8^+^ T cell response

Mice were adoptively transferred with CD45.1^+^ splenic OT-I cells (2×10^6^ cells in 200 μL PBS per mouse) via tail vein injection on day-1, and immunized through intraperitoneal injection with 2 μg of DEC-OVA, in the presence of 10 μg of control or anti-CD40 antibody per mouse. On day 6, spleen cells were harvested and treated with red blood cell lysis buffer, the single cell suspension was stained with anti-CD4 (ebioscience, clone RM4-5), anti-CD8 (Biolegend, clone 53-6.7), anti-CD45.1 (Biolegend, clone A20), anti-TCR-Vα2 (Biolegend, clone B20.1) to quantify OVA-specific OT-I CD8^+^ T cells. DAPI (Invitrogen, D3571) was used to distinguish live cells. OT-I CD8^+^ T cell is defined as CD45.1^+^CD8^+^TCR-Vα2^+^ cells (Figure S2).

### Construction and identification of *FUT8*^-/-^ CHO-K1

The 10^th^ exon of *FUT8* gene encoding the catalytic site of the fucosyltransferase 8, was selected as the knockout target. Three sgRNAs were designed (with the protospacer sequences: 5′-CAGAGTCCATGTCAGACGCA-3′, 5′-CTGATGACCCTTCTTTGTTA-3′, and 5′-TGACCCTTCTTTGTTAAAGG-3′). Three pairs of annealed sgRNA were separately ligated to the linear plasmid lentiCRISPR v2 digested by BsmBI (NEB). lentiCRISPR v2 together with pVSVg and psPAX2 were transfected into HEK293T cells to produce lentivirus. CHO-K1 cells at 80 % confluence were infected by the mixed lentivirus and cultured for 12 h and then screened with 8 μg/mL puromycin for 7 days. Single cell clone was sorted by flow cytometry and cultured for 20 days. Genome DNA of each clone was extracted, and the *FUT8* gene was PCR-amplified with primers FUT8-F (sequence: 5′-GTGCCCCCATGACTAGGGATA-3′) and FUT8-R (sequence: 5′-GCAACAAGAACCACAAGTTCCC-3′). The PCR products were applied to Sanger sequencing.

### Fc surface display

Wild-type Fc or Fc variant fragments were cloned into the mammalian cell display vector (Figure S1A) by EcoRI and NheI restriction sites. The vector was co-transfected with pMDLg/pRRE, pRSV-Rev and pCMV-VSV-G plasmids into HEK293T cells to produce lentivirus for 48 h. Cell culture supernatant containing lentivirus was applied to HEK293T, CHO-K1 or *FUT8*^-/-^ CHO-K1 and incubated for 48 h. The cells were stained with 250 nM biotinylated FcγRIIIa^F158^ or FcγRIIIa^V158^ as described above and analyzed by flow cytometry (LSR Fortessa, BD).

### Antibody N-linked oligosaccharide analysis

1% RapiGest SF was added to 15 μg samples to denature the antibodies at 90 °C for 3 min. N-linked oligosaccharides were released with Rapid PNGase F at 50 °C for 5 min before fluorescence-labeling with the RFMS (RapiFluor-MS). The labeled oligosaccharides were purified by GlycoWorks HILIC µElution and analyzed by UPLC using Waters BEH Glycan Amide column (1.7 μm, 2.1×150 mm). After sample injection, the column was eluted by solvent A, 50 nM ammonium formate (pH 4.5) and solvent B, 100% ACN (from 75% to 63% in a linear fashion) at a flow rate of 0.4 mL/min, 60 °C. The elution profile was monitored by fluorescence detection with excitation at 265 nm and emission at 425 nm. The percentage of each oligosaccharide was calculated by the peak areas.

### Molecular modeling

The crystal structures of human FcγRIIIa in complex with Fc (PDB ID: 3SGJ) and human FcγRIIb in complex with Fc (PDB ID: 3WJJ) were used in the modeling. The structures were prepared with the “Protein Preparation Wizard” in Schrödinger (2020-2) (Protein Preparation Wizard; Epik, Schrödinger, LLC, New York, NY, 2016; Impact, Schrödinger, LLC, New York, NY, 2016; Prime, Schrödinger, LLC, New York, NY, 2020.). The residues Q38, Q74, V158, Q169 on FcγRIIIa in 3SGJ and D238 on Fc in 3WJJ were mutated back to their wild-type residues respectively. Then they were refined using “Predict Side Chains” (Schrödinger Release 2020-2: Prime, Schrödinger, LLC, New York, NY, 2020) with backbone sampling. The virtual mutation calculation was performed with “Residue Scanning Calculations” in Schrödinger (Schrödinger Release 2020-2: BioLuminate, Schrödinger, LLC, New York, NY, 2020). The mutated residue and nearby residues were refined with “side-chain prediction with backbone sampling”. The residues within 6 Å were refined to handle mutations in flexible hinge region.

### Statistical analysis

Statistical analyses were performed with GraphPad Prism 8.0.1 and *p* values <0.05 were considered significant. *p ≤ 0.05. **p ≤ 0.01, ***p ≤ 0.001, ****p ≤ 0.0001.

## Results

### Outline of mammalian cell display-based platform for Fc engineering

**Figure 1:**
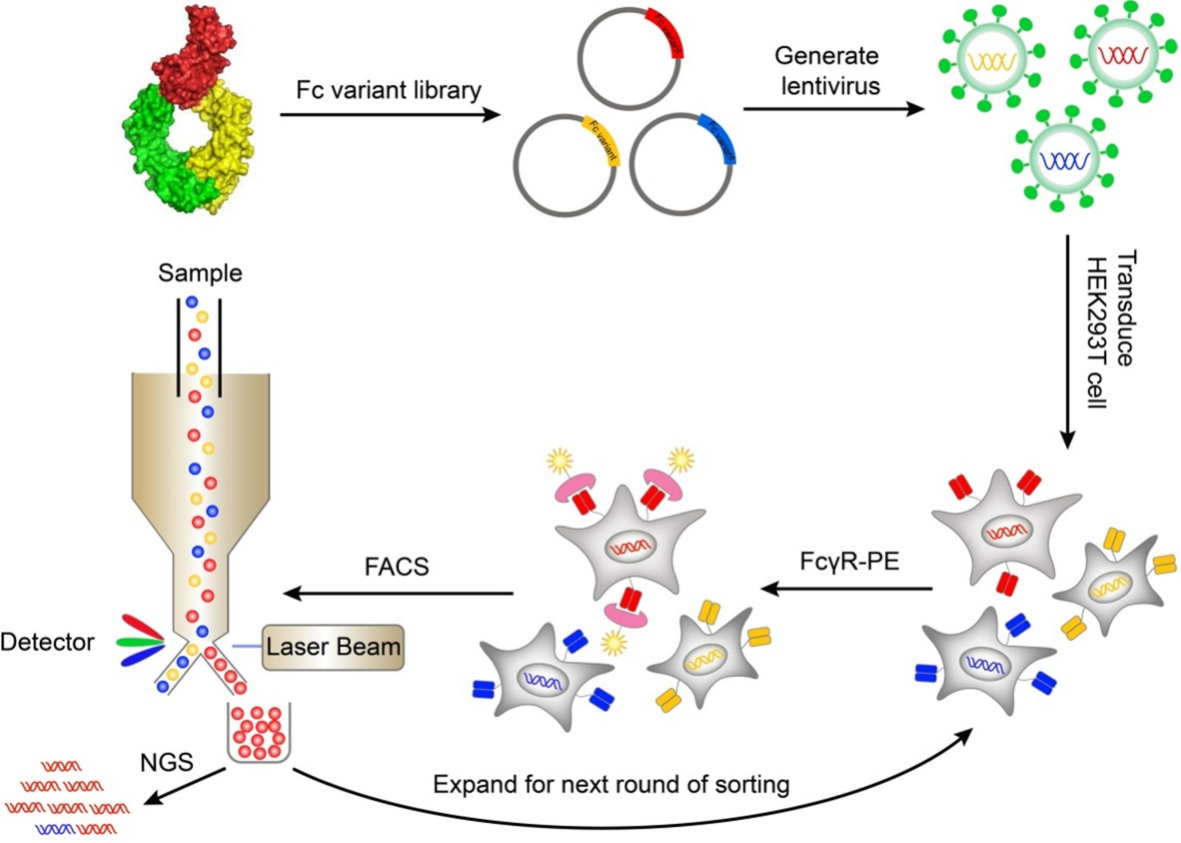
** Schematic of screening Fc variants by mammalian display.** Fc variants libraries were designed based on Fc/FcγR complex structure and cloned into lentiviral vector. The cells were transduced with Fc variant library and stained by biotinylated FcγR protein and streptavidin-PE. The cells with high FcγR binding were sorted by FACS and cultured. After iterative rounds of selection, the Fc variant genes were retrieved by PCR and the abundance of variants was quantitated by NGS. FACS, fluorescence-activated cell sorting; NGS, next generation sequencing; PE, Phycoerythrin.

A mammalian cell display system was used to express and display Fc variants on the surface of mammalian cells so that the Fc variants can be properly folded and glycosylated. The genes of Fc variants were cloned into a lentiviral vector. The lentiviral vector contained an open reading frame (ORF) encoding IL-2 signal peptide, FLAG-tag, Fc variant, and PDGFR transmembrane domain, from N terminus to C terminus (Figure S1A). The PDGFR transmembrane domain was used to anchor the Fc variant to the cell membrane. The FLAG-tag at the N terminal of Fc was used to monitor the display level of Fc. The cells were transduced with Fc variant lentiviral library and stained by biotinylated FcγR protein followed by streptavidin-PE. The cells with high FcγR binding were sorted by FACS and cultured. After multiple rounds of iteration, the enriched variants were identified by next generation sequencing (Figure 1).

To validate the function of Fc domain displayed on mammalian cell surface, HEK293T cells were infected with Fc or Fc mutant (N297A) encoding lentivirus. The cells were labeled with FITC-conjugated anti-FLAG antibody, biotinylated FcγRIIIa^F158^ and streptavidin-PE. Both wild-type Fc and Fc mutant (N297A) were displayed on cell surface at a similar level as determined by anti-FLAG antibody staining (Figure S3). The FcγR binding was retained in surface-displayed wild-type Fc, but not Fc (N297A) variant (Figure S3), a mutation lacking glycosylation site and affinity for FcγRs 23. The data demonstrated that mammalian cell display can discriminate Fc variants with different binding affinity to FcγRs and therefore can be used to screen library of Fc variants.

### Construction of Fc variant libraries

Analysis of the co-crystallization structure of Fc/FcγR complex revealed several regions of the Fc that make multiple direct contacts with its receptors (Figure S4). Presumably, altering residues on the interface can affect the affinity between Fc and FcγRs. Four loops of Fc at the interface between Fc and FcγRs (the residues of Fc within 5 Å of FcγRs) were separately mutated to construct four Fc variant libraries. Each library was designed to diversify in two random positions with each of 20 amino acids.

The library construction process is illustrated in Figure S1. We designed a pool of degenerate primers and each of primers was used to diversify two positions of the chosen Fc region. More than 10^5^ transformants were obtained from each library, which is at least 10-fold excess of the theoretical diversity to ensure 95% coverage of the theoretical diversity (Table S1). To assess the library quality, we randomly picked 3 transformed clones from each library for Sanger sequencing. As expected, most clones contained 2 amino acid substitutions which conform to the library design strategy (Table S2).

### Screening of Fc variants to enhance FcγRIIIa binding

Antibodies with high affinity to FcγRIIIa showed enhanced ADCC effect 4, 9. For this purpose, we screened the Fc libraries for variants that bind FcγRIIIa with a higher affinity.

**Figure 2:**
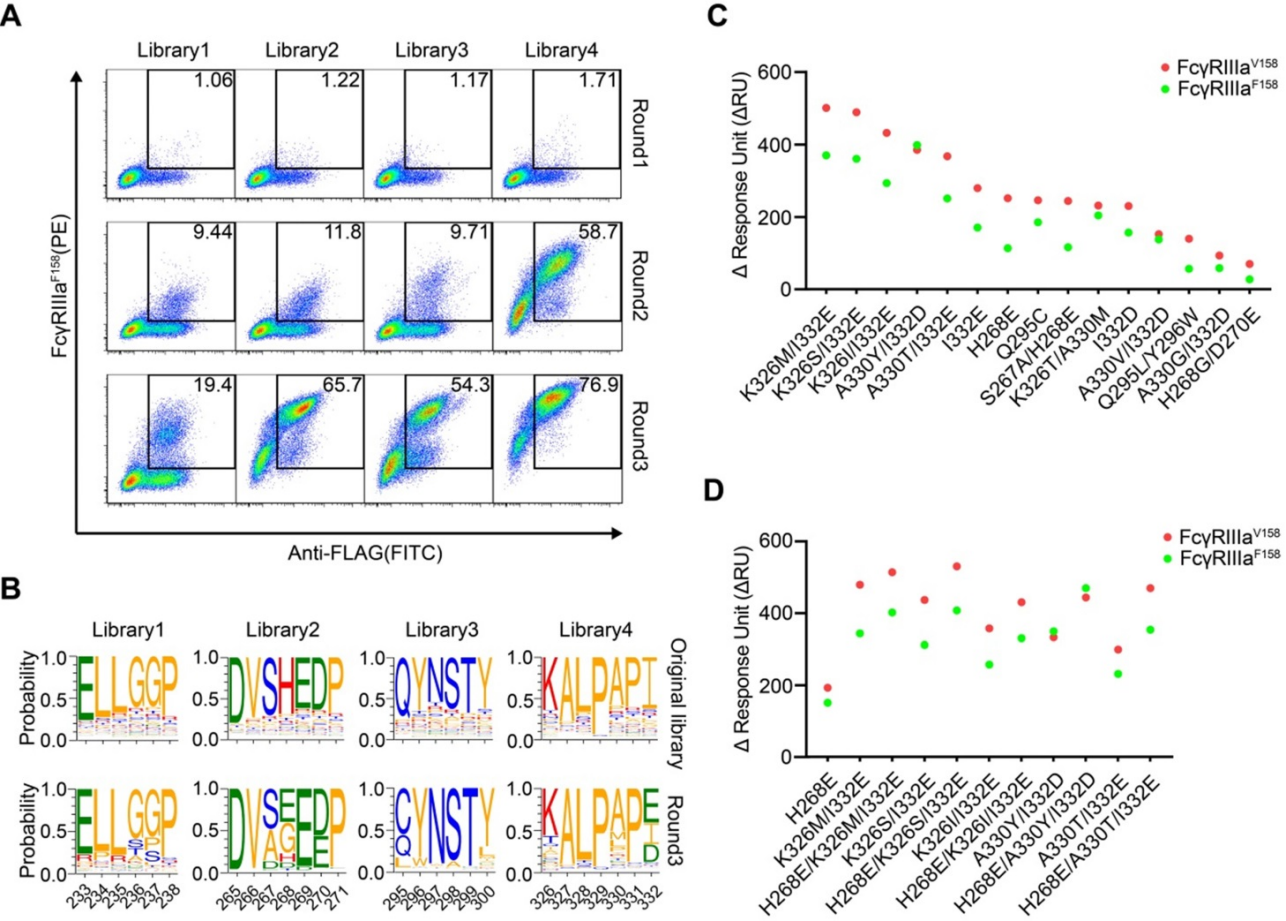
** Screening of Fc libraries for variants with enhanced FcγRIIIa binding.** (A) Enrichment of cells with the highest binding to FcγRIIIa during three rounds of selection. HEK293T cells were infected with lentiviral Fc variants libraries at low multiplicity of infection. The cells were stained with FITC conjugated anti-FLAG antibody, biotinylated FcγRIIIa and streptavidin-PE and double positively staining cells were sorted and cultured for the next round of selection. (B) The amino acid distribution at each mutation position of the original libraries and the enriched pools after the 3^rd^ round of selection. The relative frequency of each amino acid at each position is represented by the height of the corresponding symbol. (C, D) Binding of Fc variants with the selected substitutions (C) or the combination of substitutions (D) to FcγRIIIa^F158^ and FcγRIIIa^V158^ was measured by surface plasmon resonance (SPR). ΔRU is equal to the response unit of Fc variant subtracted by the response unit of wild-type Fc.

HEK293T cells were infected with lentivirus encoding Fc variant library at a low multiplicity of infection to ensure that each of infected cells expresses only one unique Fc variant. The cells were stained with FITC-conjugated anti-FLAG antibody, biotinylated FcγRIIIa and streptavidin-PE. The FITC and PE double-positive cells were sorted and cultured for the next round of selection. Three rounds of selection were performed. Cells from all four libraries showed a round-wise increase in the positively stained population. After the 3^rd^ round of sorting, all enriched cells demonstrated higher FcγRIIIa^F158^ binding (Figure 2A).

The original libraries and the enriched pools were analyzed by NGS. As shown in Figure 2B, for the original Fc variant libraries, the wild-type residue at each position made up the predominant proportion and alternative amino acid substitutions contributed to the remaining proportion. Cumulative frequency statistics of Fc variants before or after selection revealed that the diversity was remarkably reduced after selection and a subset of variants recurred frequently (Figure S5). Dozens of variants were identified over 1000 copies, which accounted for >80 % of all sequences in the enriched pools.

The large collection of NGS data also enabled us to explore the amino acid preferences after selection. The enriched non-synonymous substitutions were reasonably regarded as beneficial mutations. For library 2, H268E mutation accounted for 40.29% of enriched variants (Figure 2B). As for library 3, Q295C was observed in large proportion, accounted for 55.61% of enriched variants (Figure 2B). In library 4, I332E and I332D emerged as the predominant mutations, A330L and A330M accounted for 18.46 % of enriched variants (Figure 2B), indicating that negatively charged side chains and longer lipophilic side chains at these two positions are beneficial. Some of the identified mutations (such as I332E) had also been identified previously by protein design algorithm 9, validating the use of mammalian cell display as a functional tool for the selection of Fc variants with increased binding to FcγRs (Figure 2B).

### The enriched Fc variants exhibit improved FcγRIIIa affinity

To test whether the enriched Fc variants have enhanced binding to FcγRIIIa, we selected 15 most enriched variants according to the NGS result, and the affinities of Fc variants against FcγRIIIa^F158^ and FcγRIIIa^V158^ were determined by SPR method. As shown in Figure 2C, all selected Fc variants exhibited improved binding affinities to both FcγRIIIa^F158^ and FcγRIIIa^V158^ compared with the wild-type Fc, demonstrating the efficiency of our approach.

To further optimize the binding affinity to FcγRIIIa, we combined H268E with K326M/I332E, K326S/I332E, K326I/I332E, A330Y/I332D or A330T/I332E in the Fc variant. Combination of the substitutions provided substantial improvement in the binding affinity to both the high-affinity FcγRIIIa^V158^ and low-affinity FcγRIIIa^ F158^ allotypes, in comparison with the uncombined ones (Figure 2D).

To examine the affinity of Fc variants to different FcγRs in the context of intact antibody, the Fc variants H268E/K326M/I332E, H268E/K326S/I332E, H268E/K326I/I332E, H268E/A330Y/I332D and H268E/A330T/I332E were incorporated into rituximab. The antibodies with different Fc variants were produced and analyzed by size exclusion chromatography (SEC). They were presented as monomers and no obvious aggregation was observed (Figure S6). All the rituximab variants showed enhanced binding to both FcγRIIIa^F158^ and FcγRIIIa^V158^ and a higher FcγRIIIa/FcγRIIb binding ratio compared with wild-type Fc rituximab (Table 1 and Figure S7). The binding of the variants to human FcγRI and FcRn were also examined by SPR and no significant change was observed (Table 1 and Figure S7). We also examined the binding of the variants to C1q by ELISA. Mutation H268E/A330T/I332E abolished binding to C1q (Figure S8). By contrast, mutations H268E/K326M/I332E and H268E/K326S/I332E marginally improved C1q binding while mutations H268E/K326I/I332E and H268E/A330Y/I332D have no effect on C1q binding (Figure S8).

### Fc variants mediate enhanced ADCC

**Figure 3:**
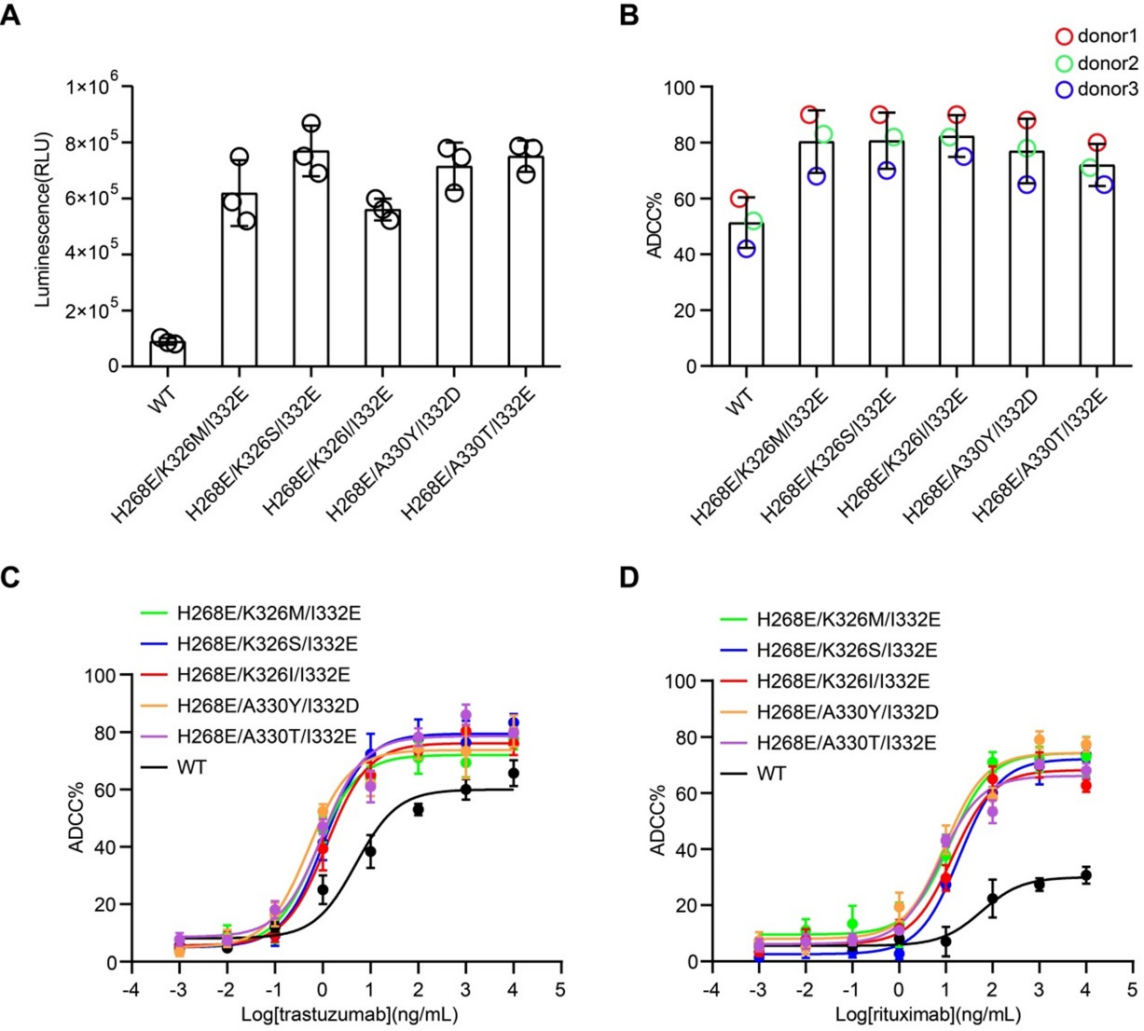
** Cell-based ADCC assays of Fc variants.** (A) Reporter cell-based assay for Fc variants. Jurkat-NFAT-FcγRIIIa reporter cell was used for monitoring the early nuclear translocation of NFAT upon ADCC induction. The reporter cells were stimulated by 1 µg well-coated Fc variants for 24 h and bioluminescent signals were measured. (B) PBMC-based ADCC assay of antibody variants. SK-BR-3 target cells were co-cultured with human PBMCs from 3 healthy donors in the presence of 1 µg/mL trastuzumab or its Fc variants. Target cell lysis was measured by the release of lactate dehydrogenase (LDH). Each color represents a different donor. (C) Concentration-dependent study of trastuzumab or its Fc variants-mediated ADCC on SK-BR-3 cells using PBMC as effector cells. (D) Concentration-dependent study of rituximab or its Fc variants-mediated ADCC on Ramos cells using PBMC as effector cells. Bars represent the mean ± SEM of three independent experiments.

Cell-based assays were performed to evaluate the capability of the Fc variants-mediated ADCC. First, we used the Jurkat-NFAT-FcγRIIIa reporter cell-based assay, in which the Fc variants were coated on a plate well to crosslink FcγRIIIa of Jurkat cells. All the Fc variants showed more potent stimulatory effect of the reporter cells than the wild-type Fc, as indicated by the higher luciferase activity (Figure 3A).

Next, the effector function of Fc variants in the context of trastuzumab and rituximab were examined by the PBMC-based ADCC assay. PBMC purified from three healthy donors were used as effector cells. HER2^+^ breast carcinoma cell line, SK-BR-3, were used as target cells for anti-HER2 trastuzumab 24. PBMC were co-cultured with SK-BR-3 in the presence of trastuzumab or its Fc variants and the release of lactate dehydrogenase (LDH) was measured. Fc variants H268E/K326M/I332E, H268E/K326S/I332E, H268E/K326I/I332E, H268E/A330Y/I332D and H268E/A330T/I332E in the context of trastuzumab demonstrated a marked increase of ADCC, as reflected in a leftward shift in the concentration response curve compared to the parental trastuzumab (Figure 3B-C). Similar enhancement of ADCC effect by the Fc variants in the context of anti-CD20 antibody rituximab has been observed in CD20^+^ Ramos cells (Figure 3D). Together, these results confirmed a positive correlation between the improved affinity for FcγRIIIa of Fc variants and enhanced ADCC effect.

### The synergistic effect of FcγRIIIa binding enhanced mutations and glycoengineering

**Figure 4:**
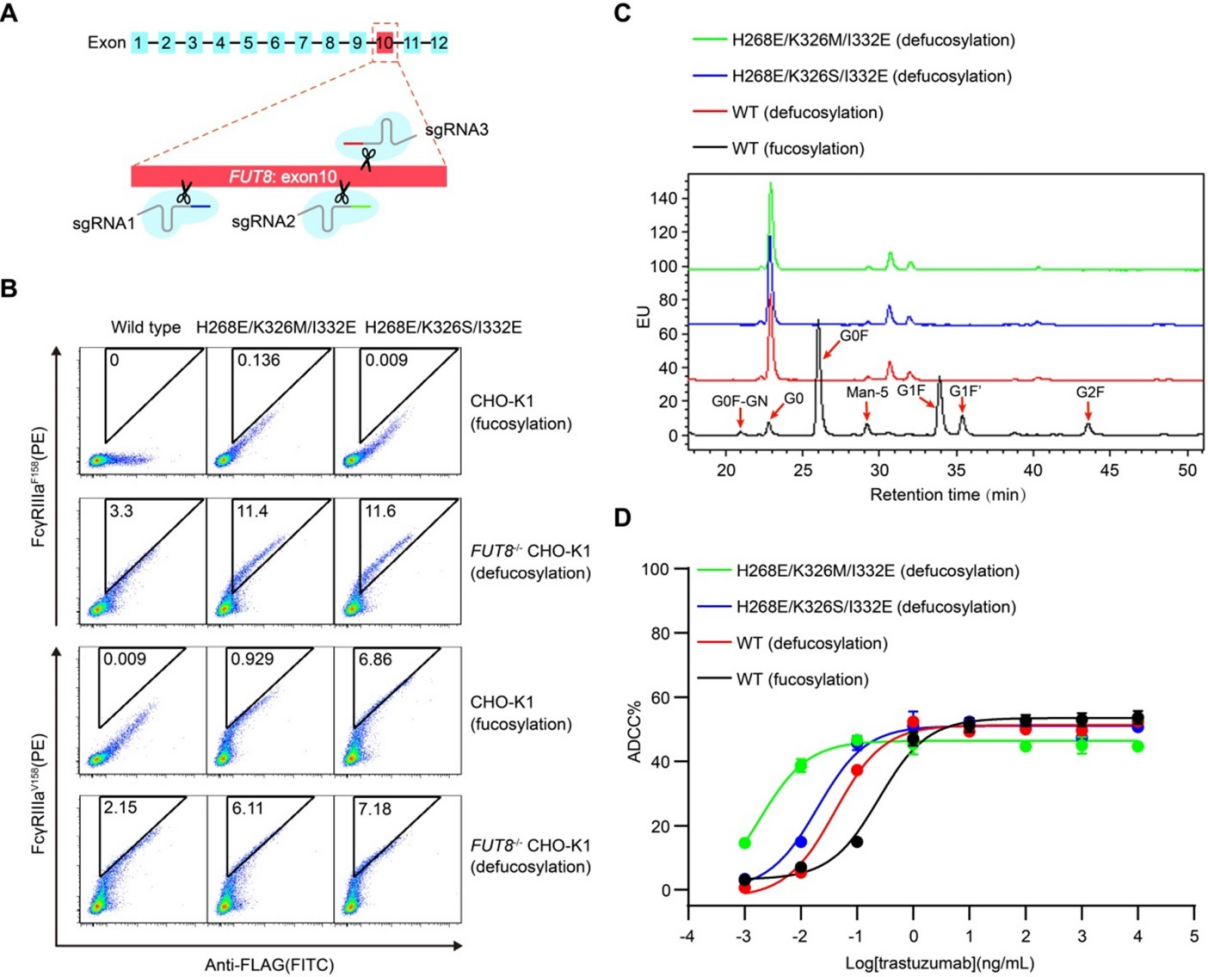
** Display of Fc or its variants by *FUT8* knockout CHO-K1 cells.** (A) Schematic of CRISPR/Cas9-mediated *FUT8* knockout in CHO-K1 cells. Three different sgRNAs were designed to target the 10^th^ exon of *FUT8* as shown in red box. (B) Wild-type Fc or Fc variants were displayed on the FUT8 expressing CHO-K1 or *FUT8*^-/-^ CHO-K1 cells. The cells were stained with FITC conjugated anti-FLAG antibody, biotinylated FcγRIIIa protein and streptavidin-PE and analyzed by flow cytometry. (C) Oligosaccharide profiles of trastuzumab variants. (D) ADCC assay of fucosylated or defucosylated trastuzumab or trastuzumab variants using SK-BR-3 as target cells and PBMC as effector cells. Bars represent the mean ± SEM of three independent experiments.

In order to test whether the mammalian cell display-based approach can be combined with glycoengineering technology, α-1,6-fucosyltransferase (FUT8) knockout CHO-K1 cell line was established using CRISPR/Cas9 technology and used to display Fc (Figure 4A and Figure S9A-B). FUT8 enzyme is responsible for the transfer of fucose to the innermost GlcNAc of Fc oligosaccharide. As expected, wild-type Fc displayed on *FUT8*^-/-^ CHO-K1 cells showed enhanced binding to FcγRIIIa compared with Fc displayed on the FUT8-expressing parental CHO-K1 cells (Figure S9B). Therefore, the mammalian cell-displayed Fc can recapitulate the enhanced FcγRIIIa binding of nonfucosylated Fc, suggesting that the mammalian cell display-based system can be used to dissect the effect of glycosylation on therapeutic proteins.

Furthermore, Fc variants with enhanced FcγRIIIa binding were also displayed on the surface of *FUT8*^-/-^ CHO-K1 cells and FUT8-expressing parental CHO-K1 cells. The defucosylated wild-type Fc exhibited higher binding to FcγRIIIa compared with the fucosylated Fc variants (Figure 4B). Nevertheless, the defucosylated Fc variants exhibited enhanced binding to both FcγRIIIa allotypes compared with the defucosylated wild-type Fc (Figure 4B), suggesting the combination of defucosylation and protein mutations had an additive effect in FcγRIIIa binding enhancement. Further, we purified trastuzumab with wild-type Fc or Fc variants from *FUT8*^-/-^ CHO-K1 cells and analyzed the oligosaccharide profiles of the antibodies. The *FUT8*^-/-^ CHO-K1 cells expressed antibodies with extremely low fucose content compared with parental CHO-K1 cells (Figure 4C and Table S3).

In agreement with the hierarchy of mammalian displayed Fc, recombinant trastuzumab with Fc of dual modification of defucosylation and mutation exhibited enhanced FcγRIIIa binding and more effective ADCC effect compared with fucose-deficient trastuzumab with wild-type Fc (Figure 4D, Table 2 and Figure S10), indicating that Fc could be further improved to increase ADCC effect.

### Screening of variants with enhanced FcγRIIb binding

**Figure 5:**
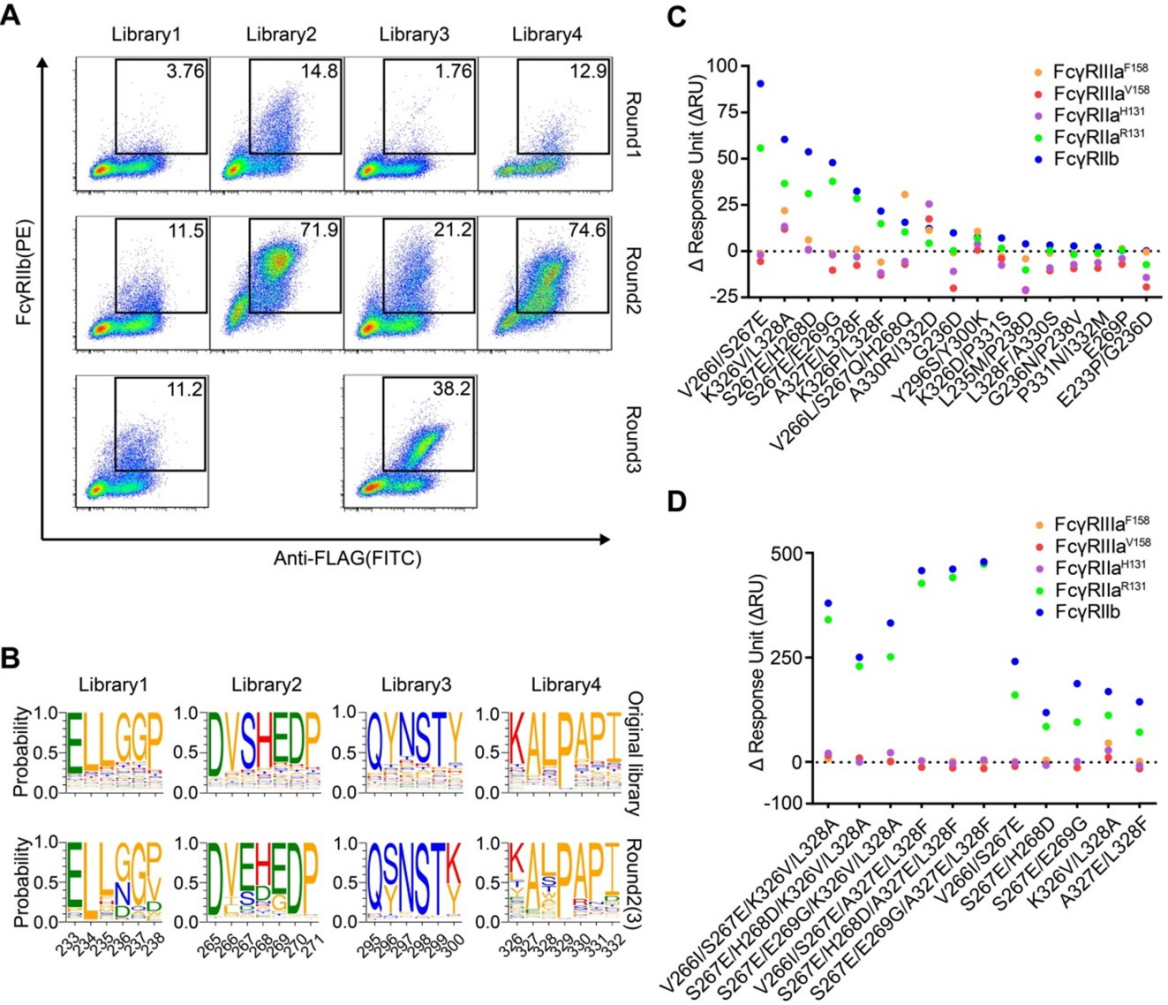
** Screening of Fc libraries for variants with enhanced FcγRIIb binding.** (A) Enrichment of cells with the highest binding to FcγRIIb. HEK293T cells were transduced with lentiviral Fc variant libraries and stained by FITC conjugated anti-FLAG antibody, biotinylated FcγRIIb and streptavidin-PE. Three rounds of selection were performed. (B) The amino acid distribution at each mutation region of the original libraries and the enriched pools after the 2^nd^ or 3^rd^ round of selection. The relative frequency of each amino acid at each position is represented by the height of the corresponding symbol. (C, D) Binding of Fc variants with the selected substitutions (C) or the combination of substitutions (D) to FcγRs was measured by surface plasmon resonance (SPR). ΔRU is equal to the response unit of Fc variant subtracted by the response unit of wild-type Fc.

FcγRIIb has been shown to drive the agonistic activity of many co-stimulatory receptor antibodies by crosslinking these antibodies 15, 16. Next, we screened the Fc libraries to identify variants that bind FcγRIIb with high affinity. After three rounds of selection, all four libraries showed a round-wise increase in positively stained cells (Figure 5A). The enriched cell pools after selection were analyzed by NGS. The diversity was remarkably reduced after selection and a subset of variants recurred frequently (Figure S11). Dozens of variants were identified over 1000 copies and the enriched mutations were regarded as beneficial mutations (Figure 5B).

### The enriched variants show higher affinity to FcγRIIb

Seventeen most enriched Fc variants by FcγRIIb were expressed, purified and all Fc variants possessed higher binding affinity to FcγRIIb than the wild-type Fc (Figure 5C). Variants L235M/P238D and G236D enhanced binding to FcγRIIb while the binding to FcγRIIa^R131^ was decreased or unchanged relative to the wild-type Fc (Figure 5C). Among these variants, S267E and L328F mutations were previously reported, while K326V/L328A was a novel variant with enhanced affinity to FcγRIIb (Figure 5C).

In order to further enhance the affinity of Fc to FcγRIIb, we generated Fc variants by combining V266I/S267E, S267E/H268D or S267E/E269G of library 2 with either K326V/L328A or A327E/L328F of library 4. As expected, the combined substitutions further enhanced Fc binding to FcγRIIb (Figure 5D). V266I/S267E/K326V/L328A was a novel variant which showed enhanced binding to FcγRIIb to a similar degree to the S267E/L328F mutation (SELF) 25 (Table 3 and Figure S12). Then P238D or G236D was introduced into variant V266I/S267E/K326V/L328A to increase the selectivity to inhibitory FcγRIIb.

We previously identified a potent FcγR-dependent CD40 agonist antibody NK003. We replaced the Fc of NK003 with variants V266I/S267E/K326V/L328A, G236D/V266I/S267E/K326V/L328A, P238D/V266I/S267E/K326V/L328A and S267E/L328F. All the variants showed enhanced binding to FcγRIIb compared with wild-type Fc NK003 (Table 3 and Figure S12). The binding of the variants to human FcγRI and FcRn were also examined by SPR and no significant changes were observed (Table 3 and Figure S12). We also examined the binding of the variants to C1q by ELISA. All the variants have no effect on C1q binding except variant V266I/S267E/K326V/L328A displayed slightly increased binding to C1q (Figure S13). Size exclusion chromatography analysis revealed that all antibodies with Fc variants existed as monomers without aggregation (Figure S14).

The variants G236D/V266I/S267E/K326V/L328A and P238D/V266I/S267E/K326V/L328A had enhanced binding to FcγRIIb and selectivity for FcγRIIb, reflected as the lower FcγRIIb/FcγRIIa^R131^ K_D_ ratio than prototype (Table 3).

### CD40 antibody variants display differential FcγRIIa and FcγRIIb- dependent agonistic activity

CD40 agonistic antibodies demonstrated encouraging efficacy in melanoma, pancreatic carcinoma and lymphoma in early clinical trials 26-28. Crosslinking of Fc by FcγRIIb expressed on tumor infiltrating immune cells is considered essential for the antitumor activity of human CD40 agonist antibodies, whereas engagement of the activating FcγRIIa inhibits this activity 22.

In order to elucidate the influence of the affinity and selectivity towards FcγRIIb of Fc variants on the agonistic activity of CD40 antibodies, activation of CD40 by antibody variants was measured on CD40 reporter cells co-cultured with HEK293T cells expressing FcγRIIb, FcγRIIa^H131^ or FcγRIIa^R131^.

**Figure 6:**
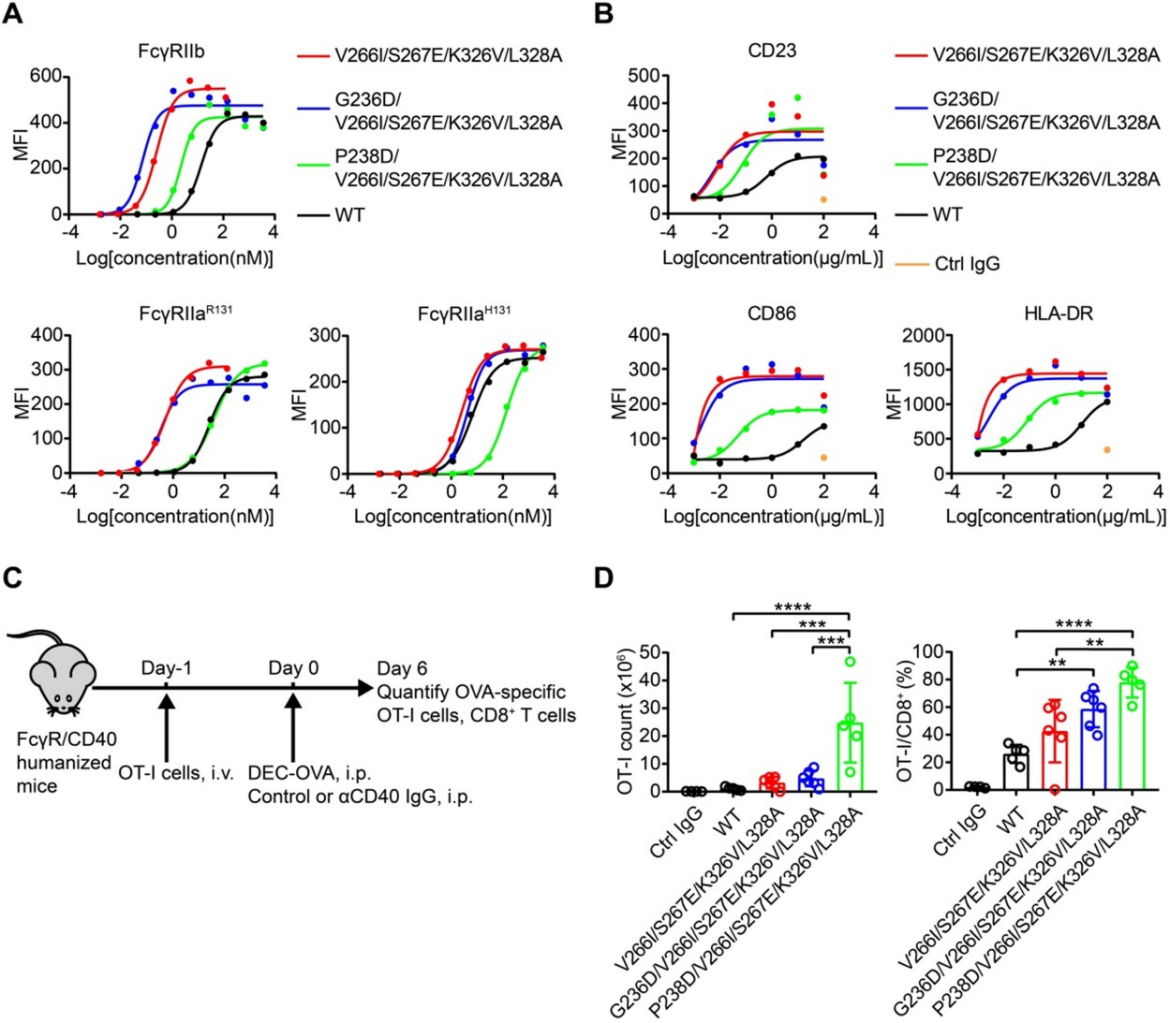
** Engineered CD40 agonist antibodies displayed divergent FcγRIIa and FcγRIIb-dependent agonistic activity.** (A) Different concentrations of CD40 agonist antibody variants were added to CD40 reporter cells in the presence of human FcγRIIb, FcγRIIa^R131^ or FcγRIIa^H131^ -expressing HEK293T cells, the activation of CD40 after 24 h stimulation was indicated by the expression of GFP of the reporter cell and analyzed by flow cytometry. (B) B cells were stimulated by different concentrations of NK003 variants and the upregulation of CD23, CD86 or HLA-DR was analyzed by flow cytometry. MFI: Mean Fluorescence Intensity. (C) Schematic of OVA-specific CD8^+^ T cell response model. FcγR/CD40-humanized mice were adoptively transferred with OT-I cells, and then immunized with DEC-OVA in the presence of CD40 antibody variants, expansion of OT-I cells was analyzed by flow cytometry. (D) Quantification of OT-I cells (left) and the percentage of OT-I cells among CD8^+^ T cells (right) as in OVA-specific CD8^+^ T cell response model. Each circle represents an individual mouse. Bars represent the mean ± SEM. ** *p* ≤0.01, *** *p* ≤0.001, **** *p* ≤0.0001; One-way ANOVA Tukey's multiple comparisons test was used.

Compared with the parental CD40 agonist antibody, the antibodies with Fc variants V266I/S267E/K326V/L328A, G236D/V266I/S267E/K326V/L328A, P238D/V266I/S267E/K326V/L328A and the S267E/L328F mutation exhibited more potent agonism in the presence of FcγRIIb-expressing cells, as reflected in a leftward shift in the curve and a smaller EC50 (Figure 6A, Figure S15 and Table S4-6).

Nevertheless, the substitution of NK003 with different Fc variants resulted in differential activation of reporter cells in the presence of HEK293T expressing FcγRIIa^H131^ or FcγRIIa^R131^ (Figure 6A, Figure S15 and Table S4-6), consistent with their different K_D_ (FcγRIIb)/K_D_ (FcγRIIa) ratios (Table 3). And the ratios of EC50 (FcγRIIb)/EC50 (FcγRIIa) of variants G236D/V266I/S267E/K326V/L328A and P238D/V266I/S267E/K326V/L328A were lower than that of variants V266I/S267E/K326V/L328A and S267E/L328F, indicating a better selectivity for FcγRIIb (Figure 6A, Figure S15 and Table S4-6).

B cells isolated from PBMC can be activated by CD40 agonist antibody, as reflected by the upregulation of activation markers such as CD23, CD86 and HLA-DR 29. In addition, B cells express FcγRIIb 30. B cell-based assay was used to study the effect of Fc variants on the agonist activity of CD40 antibodies. Consistent with the enhanced FcγRIIb binding of variants V266I/S267E/K326V/L328A, G236D/V266I/S267E/K326V/L328A and P238D/V266I/S267E/K326V/L328A, these antibodies upregulated the activation markers more than the parental CD40 agonist antibody (Figure 6B).

### The adjuvant activity of CD40 antibody variants

In order to assess whether these FcγRIIb-enhanced Fc variants impact on the *in vivo* activity of CD40 antibodies, the immunostimulatory activity of CD40 antibodies was evaluated in FcγR/CD40-humanized mice, which recapitulates the expression profile of human FcγRs (Figure 6C) 31. Mice treated with CD40 antibodies showed an increased number and percentage of OT-I cells among CD8^+^ T cells, as compared to mice treated with isotype control antibody (Figure 6D). Fc variants V266I/S267E/K326V/L328A with enhanced hFcγRIIb binding resulted in an increased T cell activation compared with the wild-type antibody (Figure 6D). G236D/V266I/S267E/K326V/L328A and P238D/V266I/S267E/K326V/L328A with enhanced selectivity for hFcγRIIb showed more potent activity in T cell activation compared with the variant V266I/S267E/K326V/L328A (Figure 6D). These data corroborated the previous finding that enhanced FcγRIIb engagement and low FcγRIIa binding is required for the optimal *in vivo* agonistic activity of CD40 antibody therapies 22.

### Molecular modeling of mutations was performed based on energy-based scoring functions

The virtual mutation calculations were performed on the amino acid substitutions that most strikingly enhanced the affinity to understand the driving force underlying enhanced affinity. We used software Schrödinger and side-chain prediction with backbone sampling as the refinement method. The approach limits the conformational sampling to 6Å around the mutation site and leaves distant regions unperturbed, based on the assumption that mutations do not induce changes in the global tertiary structure.

**Figure 7:**
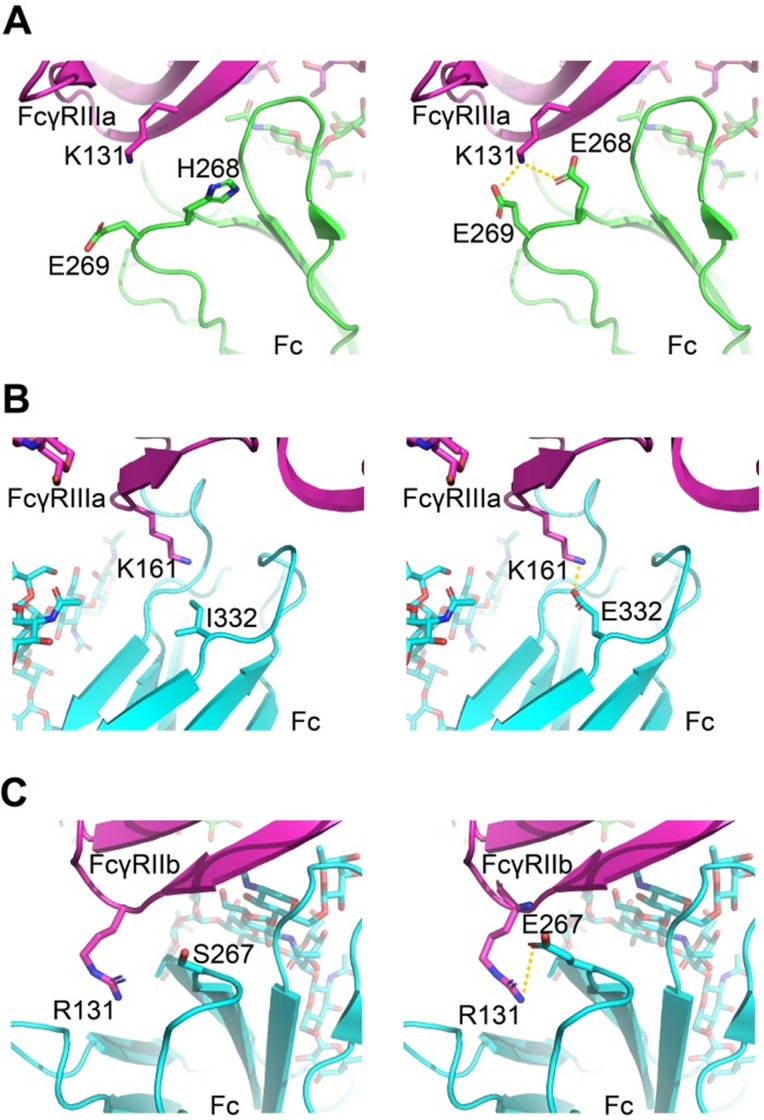
** Molecular models of Fc variant-FcγRs interactions.** (A, B) Modeling of FcγRIIIa interacting with Fc variants H268E (A) and I332E (B). (C) Modeling of FcγRIIb interacting with Fc variant S267E. The virtual mutation calculations were performed with Schrödinger software. Salt bridges are indicated by yellow dashed lines.

For FcγRIIIa/Fc binding, the structure models indicate H268E can form a salt bridge with FcγRIIIa K131, and I332E allowed a new salt interaction to be formed between Fc and amine group of the side chain of Lys161 of the receptor (Figure 7A-B). For FcγRIIb/Fc binding, the structure models indicate S267E can form a salt bridge with FcγRIIb R131 (Figure 7C). The calculated ΔΔG suggests beneficial effect on binding affinity for these mutations (Table S7).

## Discussion

In this report, we developed a mammalian cell display-based platform to engineer antibody Fc fragment. The technology offers two advantages to the current methods. First, the screening throughput is significantly increased from thousands to millions of variants so the novel Fc variants containing multiple mutations can be identified. And second, the glycosylation is retained and glycoengineering can be performed in parallel with mutagenesis.

The computational approach has become an increasingly important tool for protein engineering. The advanced algorithm was designed to search the sequence-structure space and the force field was employed to assess free energy of Fc/FcγR complex 9. A few Fc variants generated by this approach were evaluated in clinical trials. However, the computational method is, for the time being, not applicable in a high throughput manner for hundreds of thousands of mutations. Moreover, attributed to inaccuracy of the force field, there are energy discrepancies between computational predictions and experimental results. For example, the K326V/L328A mutant enhanced affinity to FcγRIIb, but it is predicted unfavorable for FcγRIIb binding (data not shown). Presumably, the experimental screening method and computational biology should be combined to optimize Fc interactions in the future.

In order to improve pharmacokinetic properties, Fc variants with enhanced neonatal Fc receptor (FcRn) binding were isolated from the Fc variant libraries using phage display 32. Glycan of Fc is not involved in its interaction with FcRn while glycosylation has a profound impact on its interaction with other FcγRs. Thus, phage display is not suitable to screen Fc variants for enhanced binding to other FcγRs. Our approach represents an authentically general method to screen Fc variants for improved binding to several types of receptors, such as FcγRs, complement C1q and FcRn.

The efficacy of several cancer targeted antibodies is thought to rely on the engagement of FcγRIIIa on NK cells to exert ADCC effect 6, 33, 34. Giles *et al*. combined NK cells expressing high-affinity FcγRIIIa^V158^ with therapeutic antibodies to enhance tumor cell killing 35. In this study, H268E/A330Y/I332D mutation enhanced ADCC in a FcγRIIIa allele-independent fashion, suggesting potential applications of this optimized Fc in patients who carry the low-binding allele of this receptor, therefore it avoids the genetic manipulation of NK cells expressing high-affinity FcγRIIIa^V158^. In this paper, we characterized only a small number of variants after screening to demonstrate practical potential of the method. Following this, one can assume to screen more variants in order to identify Fc variants with extremely high affinity to both high-affinity and low affinity FcγRIIIa allotypes. For example, the Fc variant library can be screened in the context of *FUT8^-/-^* cells to take advantage of both mutagenesis and glycoengineering, as we have proved the synergy of some mutations with glycoengineering (Figure 4). A library can also be generated to combine the mutations from different loops of Fc. Furthermore, Shields *et al.* reported that changes at residues not found at the binding interface in IgG/FcγRIIIa cocrystal structure resulted in enhanced binding to FcγRIIIa 5. Therefore, Fc mutant library generated by error-prone PCR can be used to screen for variants with enhanced binding to FcγRIIIa.

Introduction of M252Y/S254T/T256E mutations (YTE) in Fc increased human FcRn binding affinity and significantly extended the half-life and improve pharmacokinetics of antibody therapeutics. However, YTE mutations reduced FcγRIIIa binding, resulting in the reduction of ADCC activity 36. Using the method described herein, Fc variant library could build using variant M252Y/S254T/T256E as template to screen variants that improve both ADCC effect and pharmacokinetics.

Complement activity are postulated as contributors to the mechanism of action of some cancer targeting antibodies such as rituximab and daratumumab 37, 38. Engineering Fc variant with enhanced ability to recruit complement has potential for improving the next generation of cancer targeting antibodies. Recent structure analyses and mutagenesis studies revealed that complement C1q binding sites overlapped with FcγR binding sites 39, 40. Moore *et al.* found some CDC-enhancing substitutions impaired FcγR-mediated effector function due to decreased FcγRIIIa affinity 41. In order to identify variants with improved binding to both complement and FcγRs, Fc variants library can be screened by simultaneous staining with both proteins or staining one protein at alternative rounds. Comparing to the current technologies to harness the role of NK cells to treat cancer, these Fc engineered antibodies with extremely high affinity to FcγRIIIa could exhibit superior efficacy and ease of development.

While tumor-bearing mouse model is widely adopted to assess the anti-tumor efficacy of therapeutic antibodies, it is not the best choice for assessment of human FcγR-enhanced antibodies since human and mouse FcγRs own different expression pattern and divergent binding affinity to human IgG 42, 43. The replacement of mouse FcγR with human FcγR represents a reasonable animal model. Nordstrom *et al.* utilized the human FcγRIIIa transgenic mice to assess the FcγRIIIa-enhanced trastuzumab variant, which showed significant enhanced anti-tumor activity compared with wild-type one 44. Junttila *et al.* also used the FcγRIIIa-humanized mice model to compare the tumor inhibition efficacy of defucosylated trastuzumab with fucosylated trastuzumab 45. Smith *et al.* had reported the FcγR-humanized mice recapitulating hFcγR expression patterns and tested the enhanced effector function of FcγRIIIa-enhanced variant in the B cell, T cell and platelet depletion assay 46. Further, cynomolgus monkey, whose FcγRs are highly homologous to those in human, has been reported for the B cell depletion efficacy test by rituximab antibody variants 9. The above described *in vivo* model could be used to assess the efficacy of therapeutic antibodies with FcγRIIIa-enhanced Fc variants.

Although several efforts have been made to enhance Fc binding to the activating FcγRs, engineering Fc to enhance binding for the inhibitory FcγRIIb is limited. Chu *et al*. have introduced S267E/L328F substitutions into the Fc region and the resultant Fc had a 430-fold increase in binding affinity to FcγRIIb 25. However, S267E/L328F substitutions also enhanced the binding to FcγRIIa^R131^ 17. Mimoto *et al*. screened 500 variants individually and identified substitution P238D to selectively enhance FcγRIIb binding over both FcγRIIa allotypes 17. The P238D substitution was successfully isolated from our screening of millions of variants, again demonstrating the effectiveness of our platform.

CD40 antibody with Fc variant P238D/V266I/S267E/K326V/L328A displayed more potent immunostimulatory activity compared with the parental antibody, corroborating that selectively enhanced binding to FcγRIIb, but not to FcγRIIa, is required to maximize the activity of CD40 agonist antibody. To further increase the binding affinity to FcγRIIb, a second round of mutagenesis using obtained variant as a template can be conducted. The FcγRIIb high binding Fc variants may be substituted into DR4, DR5 and CD40 antibodies to improve their therapeutic efficacy 15.

Besides IgG, IgA is a very promising isotype for antibody therapeutics, since it recruits different effector cells, e.g. polymorphonuclear cells 2, 47, 48. IgM is also considered to have some inherent advantages as compared to IgG antibodies because IgM has high avidity binding to antigen and exhibits greater ability to utilize the complement dependent cytotoxicity mechanism to kill cancer cells 49. We hope the industry and academy labs can be equipped with our method to improve the activity of the emerging types of antibody therapeutics.

When we prepared the manuscript, the Coronavirus Disease 2019 (COVID-19) pandemic caused by SARS-CoV-2 emerged and spread worldwide. The spike (S) glycoprotein on the surface of SARS-CoV-2 binds human angiotensin-converting enzyme 2 (ACE2) to mediate viral entry into host cells 50. Therefore, recombinant ACE2 protein can act as a decoy receptor for S protein 51. The mammalian cell-displayed extracellular domain of ACE2 can bind S protein, enabling engineering ACE2 variants for enhanced S protein binding (Figure S16). These brief results support that our platform is a universal method for engineering glycosylated decoy receptors.

In summary, we developed an approach for screening unprecedented number of Fc variants. Fc variants with selectively enhanced FcγRIIIa or FcγRIIb binding were efficiently identified. By fine-tuning the functionality of Fc fragment, the antibodies exhibited more potent antitumor efficacy in *in vitro* or *in vivo* models comparing to the parental antibodies. The Fc engineering platform can be adopted to accelerate the development of the next generation antibody therapeutics.

## Supplementary Material

Supplementary figures and tables.

## Figures and Tables

**Table 1:** K_D_ and K_D_(FcγRIIIa)/K_D_(FcγRIIb) ratios of rituximab variants

Rituximab variants	K_D_ for FcγRI (mol/L)	K_D_ for FcγRIIb (mol/L)	K_D_ for FcγRIIIa^F158^ (mol/L)	K_D_ for FcγRIIIa^V158^ (mol/L)	K_D_ for FcγRIIa^H131^ (mol/L)	K_D_ for FcγRIIa^R131^ (mol/L)	K_D_ for FcRn pH 6 (mol/L)	K_D_ for FcRn pH 7.4 (mol/L)	K_D_(FcγRIIIa^F158^)/K_D_(FcγRIIb)	K_D_(FcγRIIIa^V158^)/K_D_(FcγRIIb)
WT	8.67E-09	2.41E-06	9.44E-07	4.03E-07	1.09E-06	1.25E-06	2.10E-08	NB	0.39	0.17
H268E/K326M/I332E	5.15E-09	1.05E-06	1.58E-07	6.91E-08	8.82E-07	7.38E-07	2.60E-08	NB	0.15	0.07
H268E/K326S/I332E	5.18E-09	1.13E-06	1.51E-07	7.47E-08	8.14E-07	8.03E-07	2.50E-08	NB	0.13	0.07
H268E/K326I/I332E	6.11E-09	1.69E-06	3.11E-07	1.43E-07	1.95E-06	1.21E-06	2.57E-08	NB	0.18	0.08
H268E/A330Y/I332D	5.04E-09	1.47E-06	9.40E-08	6.06E-08	5.26E-07	6.8E-07	2.19E-08	NB	0.06	0.04
H268E/A330T/I332E	5.64E-09	9.06E-07	1.56E-07	6.11E-08	6.27E-07	5.75E-07	2.15E-08	NB	0.17	0.07

NB, no measurable binding.

**Table 2:** K_D_ and K_D_(FcγRIIIa)/K_D_(FcγRIIb) ratios of glycoengineered trastuzumab variants

Trastuzumab variants	K_D_ for FcγRIIb (mol/L)	K_D_ for FcγRIIIa^F158^ (mol/L)	K_D_ for FcγRIIIa^V158^ (mol/L)	K_D_ for FcγRIIa^H131^ (mol/L)	K_D_ for FcγRIIa^R131^ (mol/L)	K_D_(FcγRIIIa^F158^)/K_D_(FcγRIIb)	K_D_(FcγRIIIa^V158^)/K_D_(FcγRIIb)
H268E/K326S/I332E (defucosylation)	1.46E-06	2.62E-09	1.36E-09	6.69E-07	3.69E-07	0.0018	0.0009
H268E/K326M/I332E (defucosylation)	1.03E-06	3.55E-09	1.71E-09	1.04E-06	4.20E-07	0.0034	0.0017
WT (defucosylation)	1.78E-06	4.65E-08	1.23E-08	2.09E-06	1.46E-06	0.0261	0.0069
WT (fucosylation)	1.24E-06	1.74E-07	3.85E-08	1.00E-06	1.50E-06	0.1403	0.0310

**Table 3:** K_D_ and K_D_(FcγRIIb)/K_D_(FcγRIIa) ratios of NK003 variants

NK003 variants	K_D_ for FcγRI (mol/L)	K_D_ for FcγRIIb (mol/L)	K_D_ for FcγRIIIa^F158^ (mol/L)	K_D_ for FcγRIIIa^V158^ (mol/L)	K_D_ for FcγRIIa^H131^ (mol/L)	K_D_ for FcγRIIa^R131^ (mol/L)	K_D_ for FcRn pH 6 (mol/L)	K_D_ for FcRn pH 7.4 (mol/L)	K_D_(FcγRIIb)/ K_D_(FcγRIIa^H131^)	K_D_(FcγRIIb)/ K_D_(FcγRIIa^R131^)
WT	1.04E-08	6.26E-06	2.12E-06	5.95E-07	1.39E-06	2.29E-06	3.77E-08	NB	4.50	2.73
V266I/S267E/ K326V/L328A	9.43E-09	3.69E-08	8.16E-07	4.14E-07	7.65E-07	2.11E-08	5.32E-08	NB	0.05	1.75
G236D/ V266I/S267E/ K326V/L328A	1.84E-08	1.82E-08	4.66E-06	4.98E-06	1.40E-06	1.94E-08	5.40E-08	NB	0.01	0.94
P238D/ V266I/S267E/ K326V/L328A	1.78E-08	1.08E-06	WB	NB	NB	1.98E-06	5.86E-08	NB	/	0.55
S267E/L328F	8.78E-09	2.39E-08	4.24E-06	5.22E-06	1.45E-06	1.30E-08	3.91E-08	NB	0.02	1.84

NB, no measurable binding; WB, weak binding.

## Abbreviations

ADCC: antibody-dependent cellular cytotoxicity
FcγRs: Fc gamma receptors
FcRn: neonatal Fc receptor
FITC: fluorescein isothiocyanate
FACS: fluorescence-activated cell sorting
i.v.: intravenous injection
i.p.: intraperitoneal injection
K_D_: equilibrium dissociation constant
LDH: lactate dehydrogenase
MFI: mean fluorescence intensity
MOI: multiplicity of infection
NGS: next generation sequencing
ORF: open reading frame
PBMCs: peripheral blood mononuclear cells
PE: phycoerythrin
RU: response unit
SPR: surface plasmon resonance
SEC: size exclusion chromatography
