# Supplementary material for: A general Fc engineering platform for the next generation of antibody therapeutics
Source: Theranostics. 2021 Jan 1;11(4):1901–17. doi: 10.7150/thno.51299 (PMC7778609; doi:10.7150/thno.51299)
Supplement: Supplementary file 1 — Supplementary figures and tables. [file thnov11p1901s1.pdf]

Supplementary Materials

A general Fc engineering platform for the next generation of antibody therapeutics

Da Chen[1], Yingjie Zhao[2,3], Mingyu Li[1], Hang Shang[1], Na Li[1], Fan Li[1], Wei Wang[4], Yuan Wang[1], Ruina Jin[1], Shiyu Liu[1], Xun Li[5], Shan Gao[1], Yujie Tian[1], Ruonan Li[1], Huanhuan Li[1], Yongyan Zhang[1], Mingjuan Du[4], Youjia Cao[1], Yan Zhang[2,3], Xin Li[1], Yi Huang[6], Liaoyuan A. Hu[5*], Fubin Li[2,3*], Hongkai Zhang[1,4*]

1. State Key Laboratory of Medicinal Chemical Biology and College of Life Sciences, Nankai University, 94 Weijin Road, Tianjin, 300071, China.
2. Shanghai Institute of Immunology, Faculty of Basic Medicine, Shanghai Jiao Tong University School of Medicine, Shanghai 200025, China.
3. Key Laboratory of Cell Differentiation and Apoptosis of Chinese Ministry of Education, Shanghai Jiao Tong University School of Medicine, Shanghai 200025, China.
4. Shanghai Institute for Advanced Immunochemical Studies, ShanghaiTech University, Shanghai, 201210, China.
5. Amgen Research, Amgen Biopharmaceutical R&D (Shanghai) Co., Ltd, Shanghai, 201210, China.
6. Department of Analytical Science, Zhenge Biotech, Shanghai, 201318, China.

[*]To whom correspondence should be addressed. E-mail:
hongkai@nankai.edu.cn; fubin.li@sjtu.edu.cn; liaoyuan@amgen.com

Supplementary methods

ACE2 surface display

The extracellular domain of ACE2 was PCR-amplified from the cloning plasmid (Sino biological) and inserted into the mammalian cell display vector (Figure S1A) by EcoRI and NheI restriction sites. The vector was co-transfected with pMDLg/pRRE, pRSV-Rev and pCMV-VSV-G plasmids into HEK293T cells. After 48 h of culture, supernatant containing lentivirus was applied to HEK293T cells, followed by 48 h of culture. HEK293T cells displaying ACE2 were stained with 250 nM SARS-CoV-2 Spike S1 protein with mouse Fc tag (Spike S1-mFc, Sinobiological) at 4 ℃ for 30 min. After washing three times with ice-cold FACS buffer, the cells were incubated with Alexa Fluor 647 conjugated rabbit anti-mouse IgG Fc antibody (1:200 dilution, Jackson ImmunoResearch) and ANTI-FLAG® M2-FITC antibody (1:200 dilution, SIGMA), and incubated at 4 ℃ for 30 min. After final washing, cells were analyzed by flow cytometry (LSR Fortessa, BD).

C1q binding ELISA

Three-fold serially dilution of rituximab or NK003 was immobilized on high binding 96-well plates overnight at 4 ℃. The wells were blocked with 100 µL PBST/3% BSA for 1 h followed by incubation with human C1q protein (5 µg/mL, Sigma-Aldrich) for 2 h at room temperature. After washing three times with PBST, the bound C1q was detected using HRP conjugated sheep anti-C1q antibody (1:500 dilution, Abcam) and ABTS substrate (Thermo Fisher Scientific). The OD value at 405 nm was measured by a plate reader (Molecular Devices).

Supplementary tables

Table S1. Capacity of four Fc variant libraries.

	Theoretical diversity	Library diversity
Library1	1.5×10^{4}	2×10^{5}
Library2	2.1×10^{4}	1.4×10^{5}
Library3	1.5×10^{4}	2×10^{5}
Library4	1.5×10^{4}	1.6×10^{5}

Table S2. Representative Sanger sequencing results of four Fc libraries.

Library1	Library2	Library3	Library4
**ELLGGP**	**DVSHEDP**	**QYNSTY**	**KALPAPI**
*K*Y**	**KA***	***L*N	VFP***M
*H*K**	****NQ*	M****T	Y*R****
****KN	**TE***	*****S	S*T****

Capital letters in bold, wild type sequences; capital letters, mutated amino acids; black asterisk, unchanged amino acids; red asterisk, stop codon.

Table S3. Oligosaccharide composition of trastuzumab variants.

Trastuzumab variants	G0F-GN%	G0%	G0F%	Man-5%	G1F%	G1F´%	G2F%	Others%
H268E/K326M/I332E (defucosylation)	0.1	64.3	0.1	2.7	0.0	0.0	0.0	32.8
H268E/K326S/I332E (defucosylation)	0.1	64.0	0.1	2.7	0.0	0.0	0.0	33.1
WT (defucosylation)	0.1	64.0	0.1	2.6	0.0	0.0	0.0	33.2
WT (fucosylation)	1.2	4.6	42.9	4.3	24.1	8.6	5.4	8.9

Table S4. EC50 and EC50 ratios of NK003 variants in CD40 reporter cell assay as in Figure 6A.

Variants	EC50(nM)				
	FcγRIIb	FcγRIIa^{H131}	FcγRIIa^{R131}	FcγRIIb/ FcγRIIa^{H131}	FcγRIIb/ FcγRIIa^{R131}
WT	13.81	6.49	25.91	2.13	0.53
V266I/S267E/K326V/L328A	0.26	2.55	0.50	0.10	0.52
G236D/V266I/S267E/K326V/L328A	0.07	4.27	0.34	0.02	0.21
P238D/V266I/S267E/K326V/L328A	2.28	143.00	39.25	0.02	0.06

Table S5. EC50 and EC50 ratios of NK003 variants in CD40 reporter cell assay after 6 h stimulation as in Figure S15.

Variants	EC50 (nM)				
	FcγRIIb	FcγRIIa^{H131}	FcγRIIa^{R131}	FcγRIIb/ FcγRIIa^{H131}	FcγRIIb/ FcγRIIa^{R131}
WT	7.76	0.75	7.95	10.34	0.98
V266I/S267E/K326V/L328A	0.20	0.86	0.32	0.23	0.63
G236D/V266I/S267E/K326V/L328A	0.15	1.95	0.39	0.08	0.39
P238D/V266I/S267E/K326V/L328A	1.80	68.25	8.53	0.03	0.21
S267E/L328F	0.06	0.44	0.06	0.13	0.96

Table S6. EC50 and EC50 ratios of NK003 variants in CD40 reporter cell assay after 24 h stimulation as in Figure S15.

Variants	EC50 (nM)				
	FcγRIIb	FcγRIIa^{H131}	FcγRIIa^{R131}	FcγRIIb/ FcγRIIa^{H131}	FcγRIIb/ FcγRIIa^{R131}
WT	11.08	1.14	8.29	9.74	1.34
V266I/S267E/K326V/L328A	0.47	0.68	0.66	0.69	0.71
G236D/V266I/S267E/K326V/L328A	0.42	1.06	0.76	0.40	0.55
P238D/V266I/S267E/K326V/L328A	3.05	73.75	8.89	0.04	0.34
S267E/L328F	0.20	0.58	0.23	0.34	0.85

Table S7. ΔΔG between Fc and Fc variants.

Mutants	Delta Affinity (kcal/mol)
H268E	-13.249
I332E	-2.232
S267E	-9.965

Supplementary figures

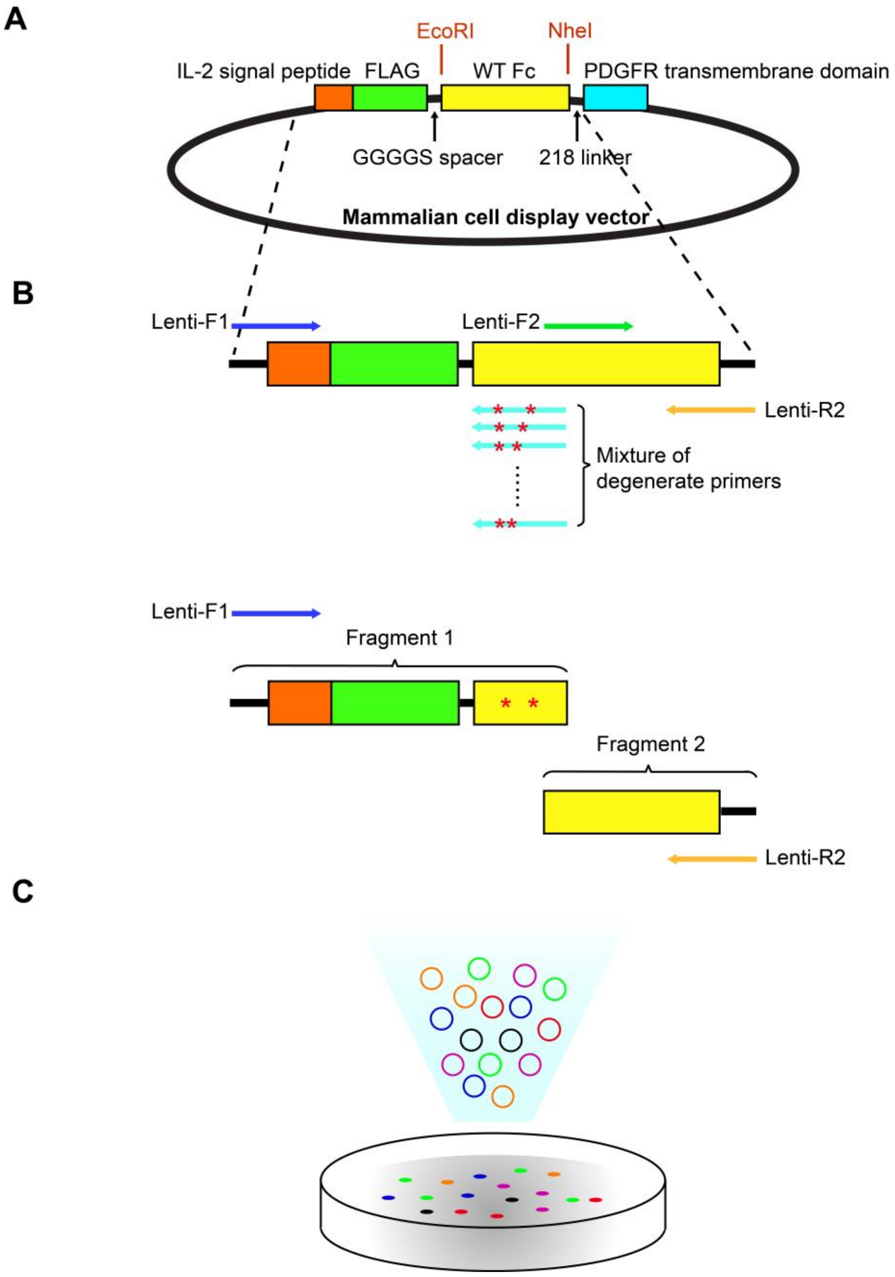

Figure S1. Schematic of Fc library construction process. (**A**) Illustration of the mammalian cell display vector. (**B**) Two fragments of Fc were PCR-amplified. Two amino acids were randomly diversified in fragment 1. Asterisks indicate mutations introduced by degenerate primers. Two fragments were combined by overlap PCR, digested by EcoRI and NheI and inserted into the mammalian cell display vector. (**C**) The plasmid pool was transformed into competent cells and the transformants were collected.

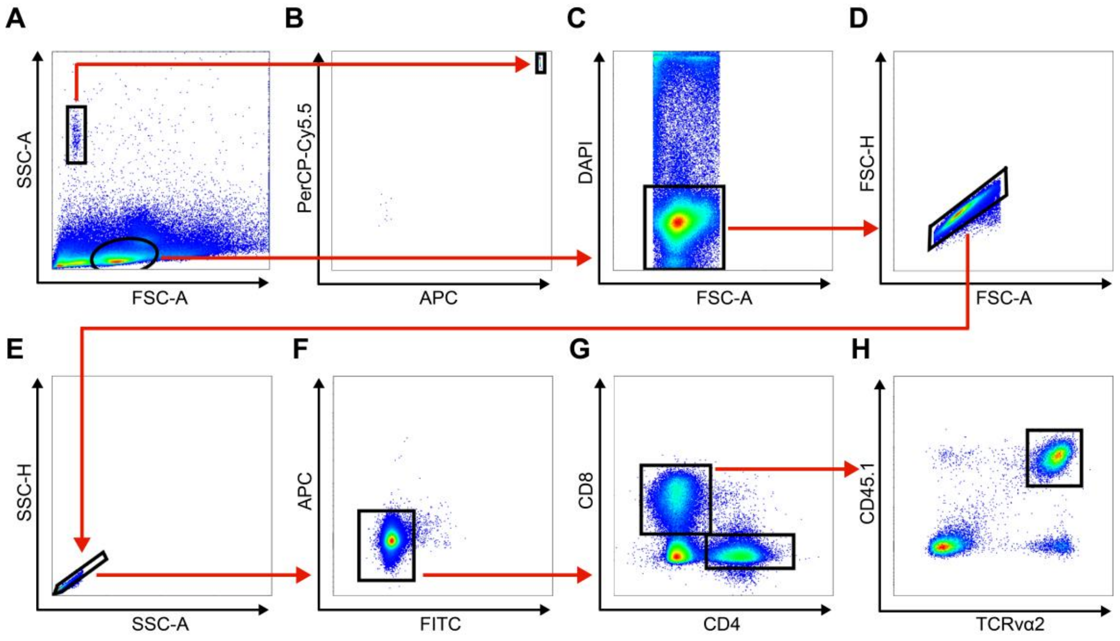

Figure S2. Gating strategy for OVA-specific CD8$^+$ T cell as in Fig. 6D. Lymphocytes were gated in the elliptical gate (**A**). The rectangle gates (**A**, **B**) were the quantitative beads added to the spleen cells for total cell number calculation. DAPI was used to exclude dead cells (**C**). FSC and SSC were used to select single cell (**D**, **E**). FITC and APC were non-staining channels to exclude non-specific fluorescence (**F**). OVA-specific CD8$^+$ T cells were defined as CD8$^+$CD4$^-$CD45.1$^+$TCRVα2$^+$ (**G**, **H**).

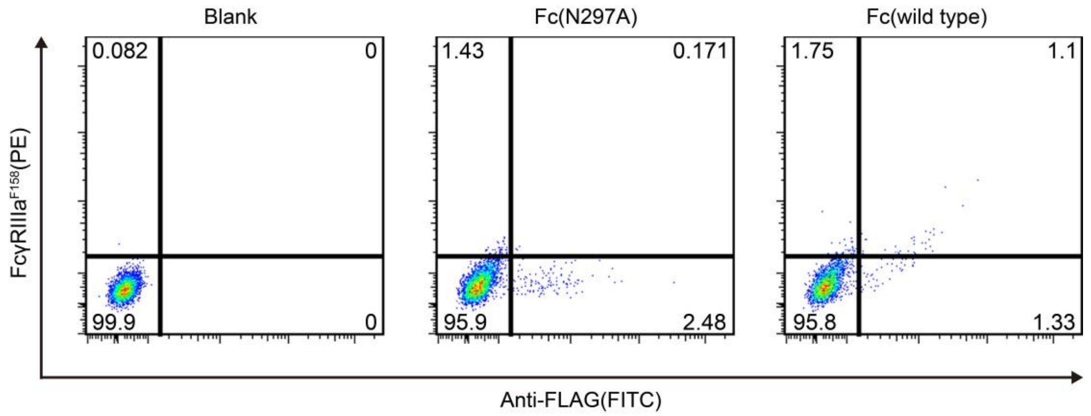

Figure S3. Display of functional Fc on HEK293T cell. HEK293T (left), HEK293T displaying Fc N297A variant (middle) or wile-type Fc (right) was stained with FITC-conjugated anti-FLAG antibody, biotinylated FcγRIIIa^{F158} and streptavidin-PE. The stained cells were analyzed by flow cytometry.

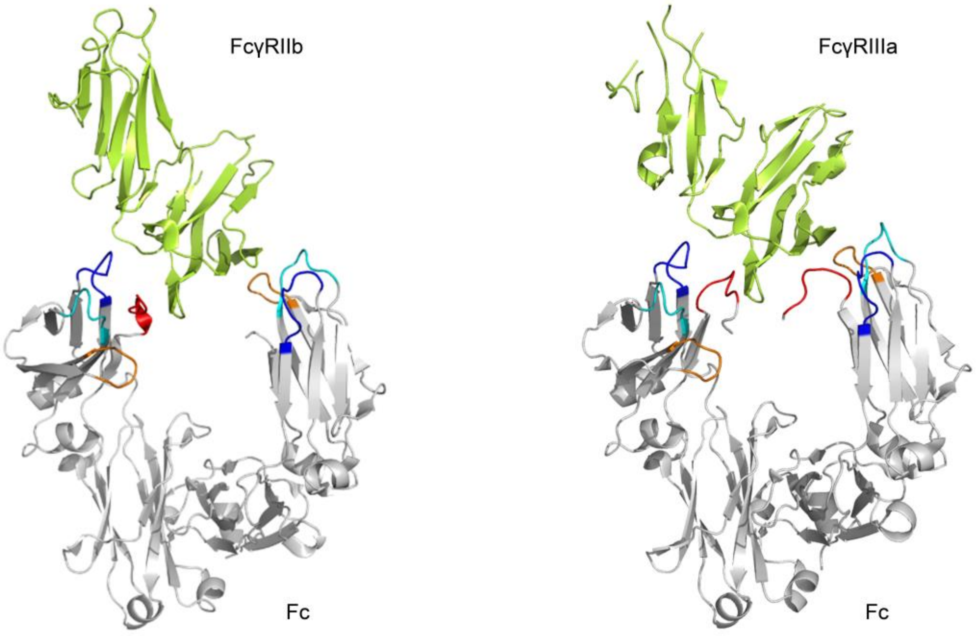

Figure S4. Structure of Fc/FcγR complexes. Fc/FcγRIIb complex structure (PDB ID 3WJJ, left) and Fc/FcγRIIIa complex structure (PDB ID 5XJE, right) were visualized by Pymol, and Fc regions corresponding to Fc variant library 1 to library 4 were colored in red, cyan, orange and blue, respectively.

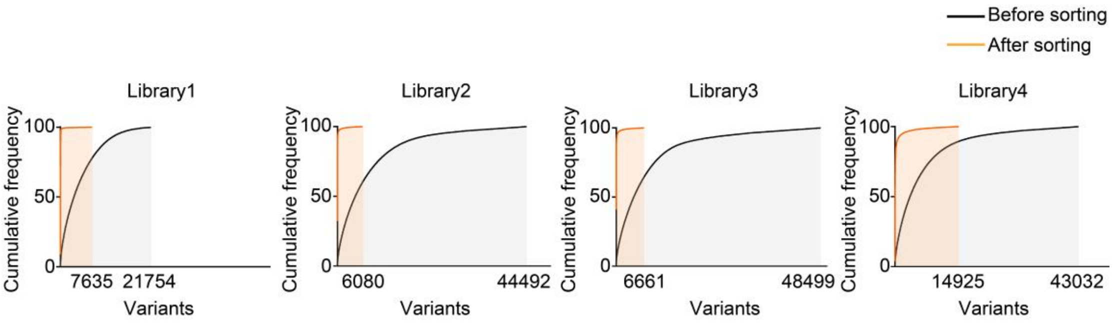

Figure S5. Cumulative frequency curve of Fc variants before or after FcγRIIIa selection. Fc variant genes were recovered from cells of the original libraries (gray) or libraries after the 3rd round of selection (orange). The abundance of variants was analyzed by NGS. The variants were depicted in X axis and the sum of the probabilities of all variants before a specific variant in X axis was depicted in Y axis.

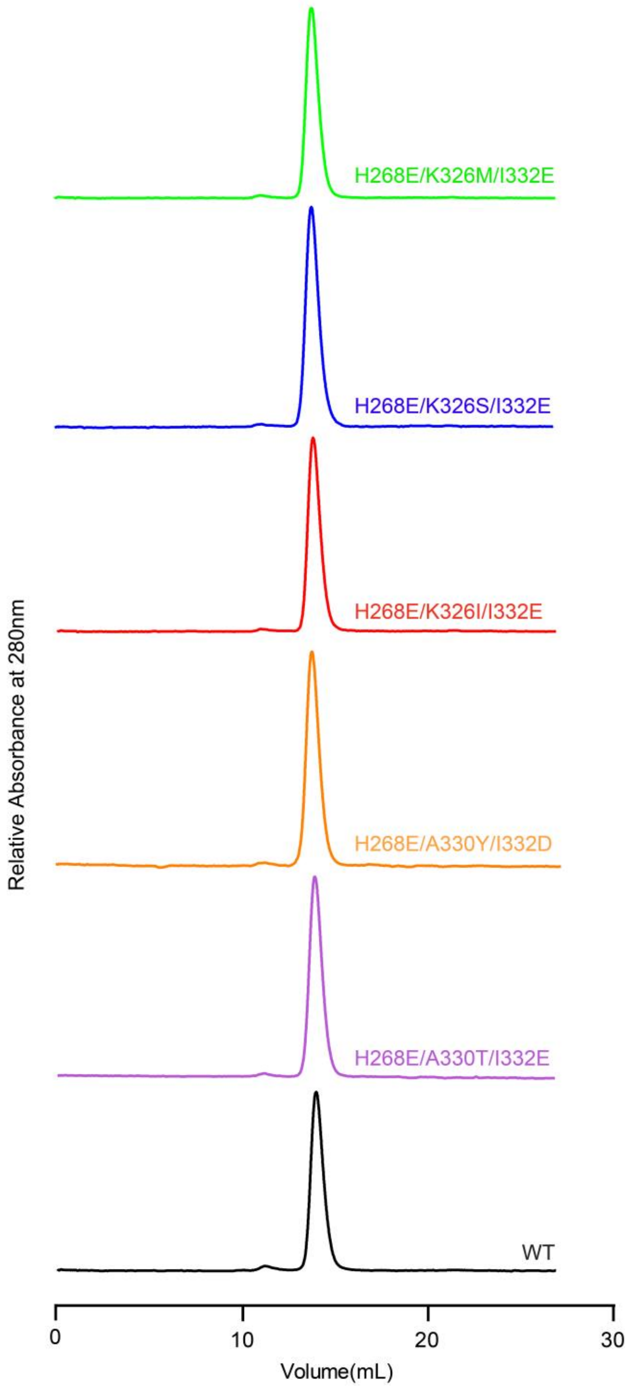

Figure S6. **Analysis of rituximab variants by size exclusion chromatography.**

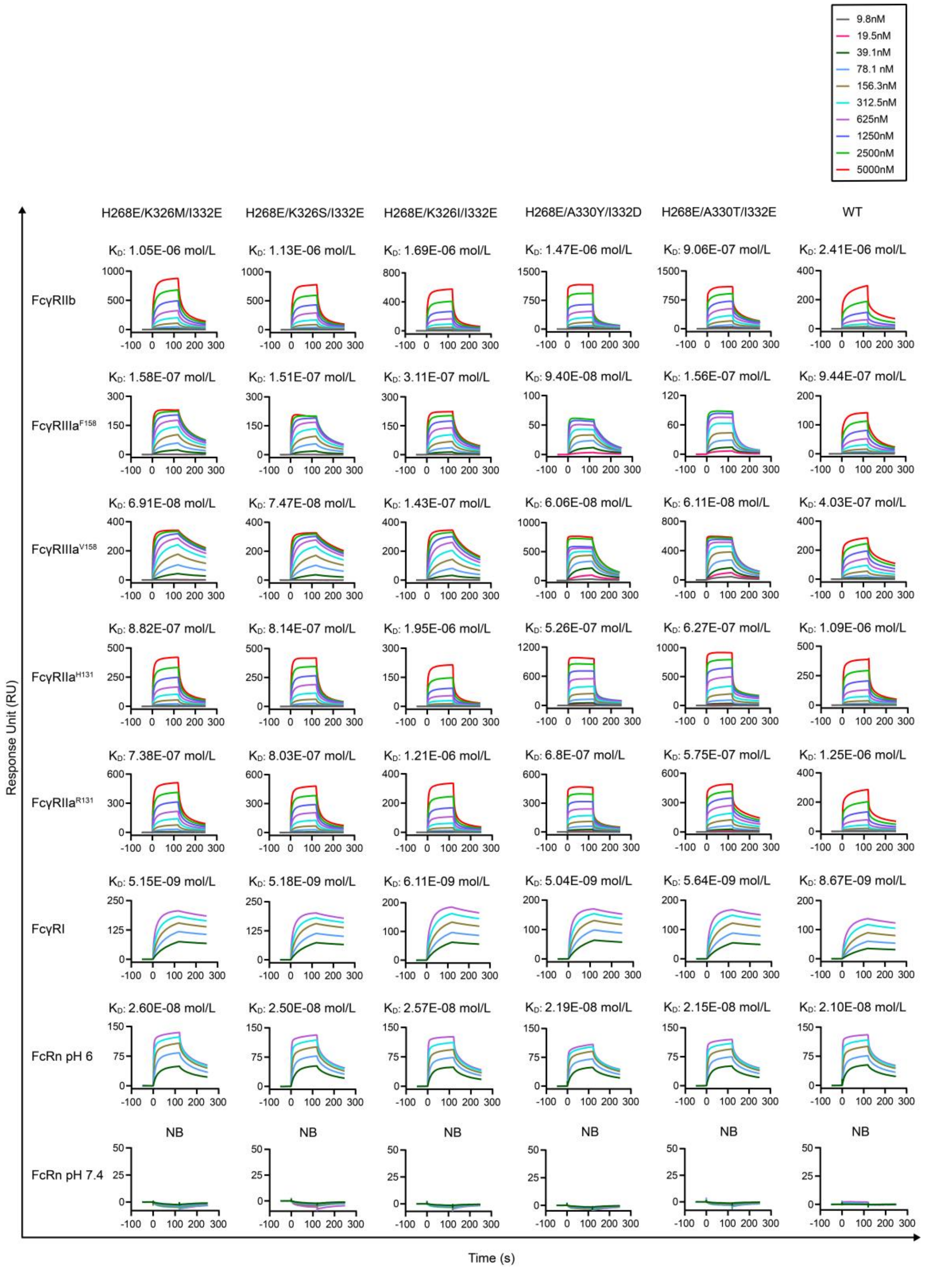

Figure S7. Surface plasmon resonance analysis of rituximab variants.

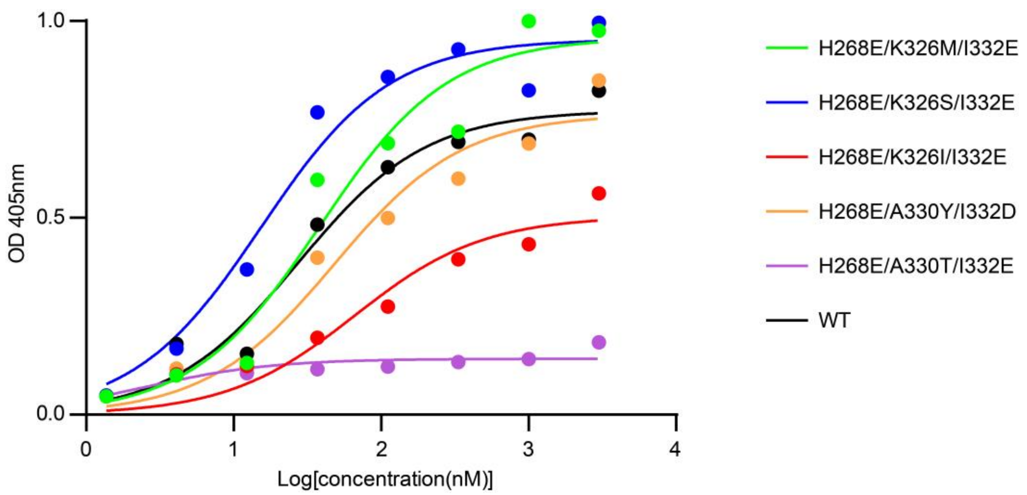

Figure S8. Binding of the human complement component C1q to rituximab variants by ELISA.

A

Both alleles genotypes of *FUT8*$^{-/-}$ CHO-K1 clones

```
Wild-type    AGAGTGTATCTGGCCACTGATGACCCTTCTTTGTTAAAGGAGGCAAAGACAAAGTAAGT
clone7-A     AGAGTGTATCTGGCCACTGAT--------------------------------------
clone7-B     AGAGTGTATCTGGCCACTGAT--------------------------------------
clone9-A     AGAGTGTATCTGGCCACTGAT--------------------------------------
clone9-B     AGAGTGTATCTGGCCACTGAT--------------------------------------
clone28-A    AGAGTGTATCTG--------------------------------CAAAGACAAAGTAAGT
clone28-B    AGAGTGTATCTGGCCACTGATGACAAAGTAAGT--------------------------
```

B

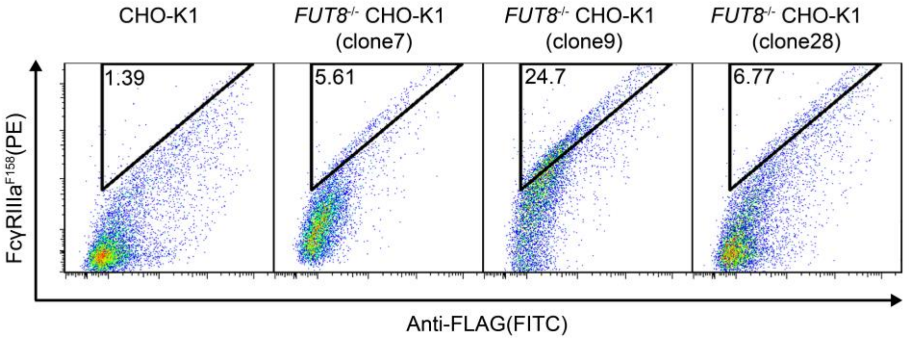

Figure S9. Establishment of *FUT8*$^{-/-}$ CHO-K1 cells. (**A**) Genotyping result of three *FUT8*$^{-/-}$ CHO-K1 clones. (**B**) Fc displayed on parental CHO-K1 or *FUT8*$^{-/-}$ CHO-K1 was stained with FITC conjugated anti-FLAG antibody, biotinylated FcγRIIIa and streptavidin-PE and analyzed by flow cytometry.

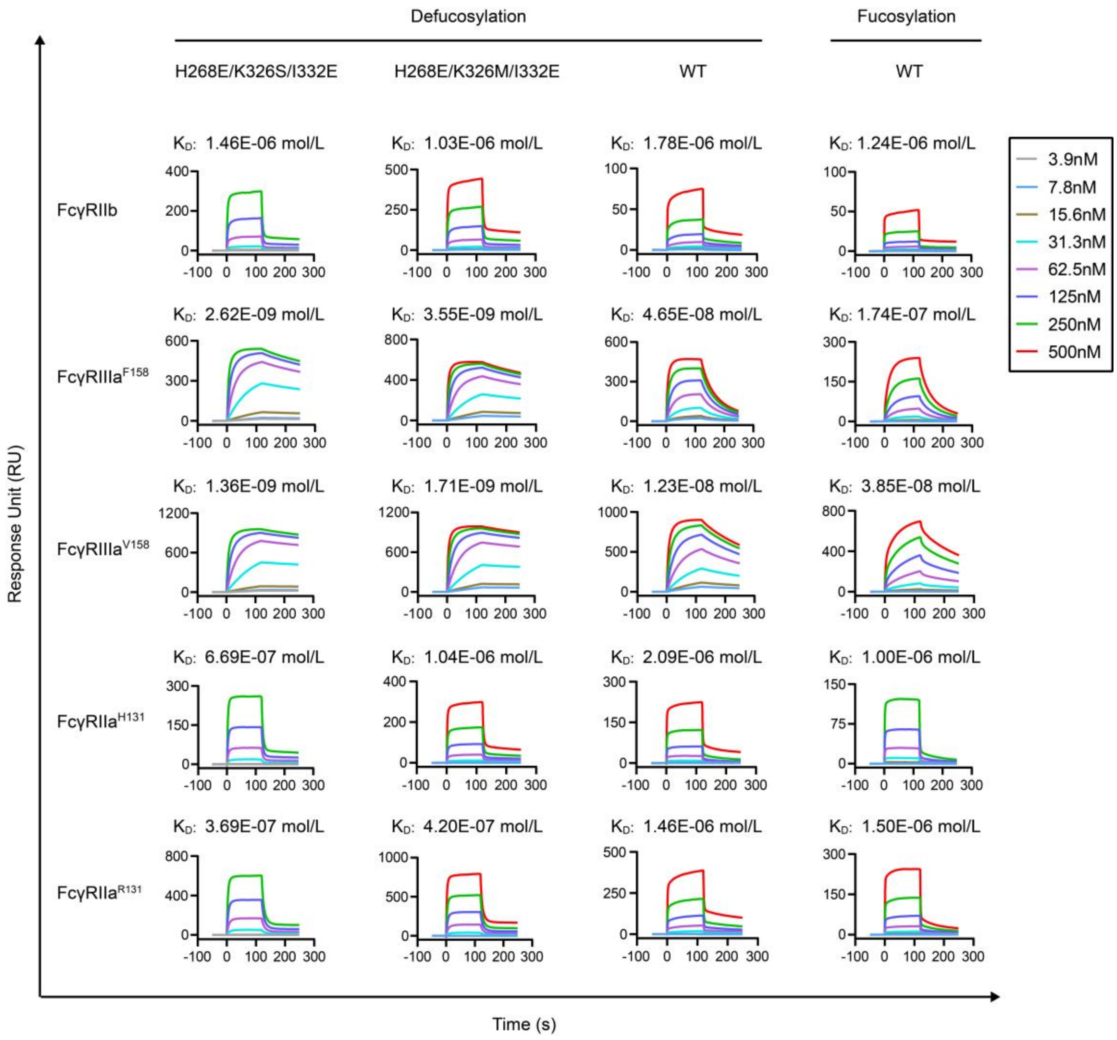

Figure S10. Surface plasmon resonance analysis of defucosylated and fucosylated trastuzumab variants.

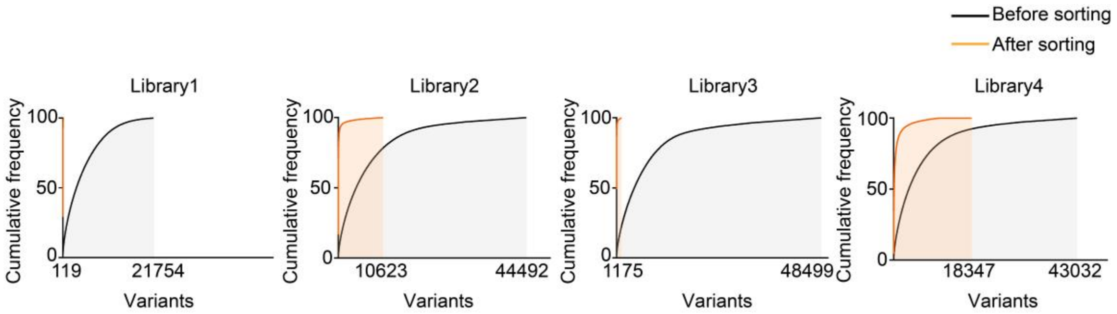

Figure S11. Cumulative frequency curve of Fc variants before or after FcγRIIb selection. Fc variant genes were recovered from cells of the original libraries (gray) or the enriched pools after the 2nd or 3rd round of selection (orange). The abundance of variants was analyzed by NGS. The variants were depicted in X axis and the sum of the probabilities of all variants before a specific variant in X axis was depicted in Y axis.

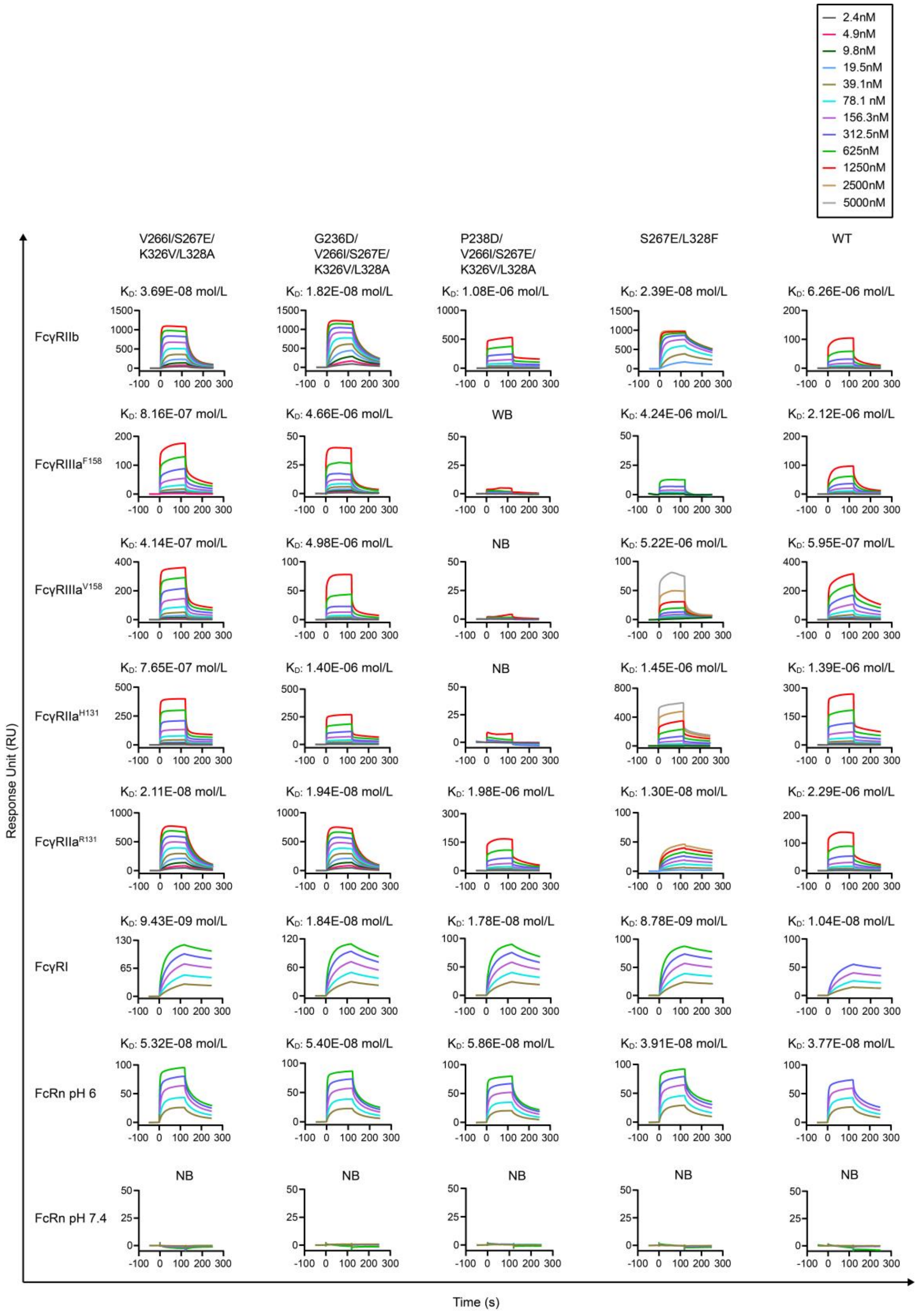

Figure S12. Surface plasmon resonance analysis of NK003 variants.

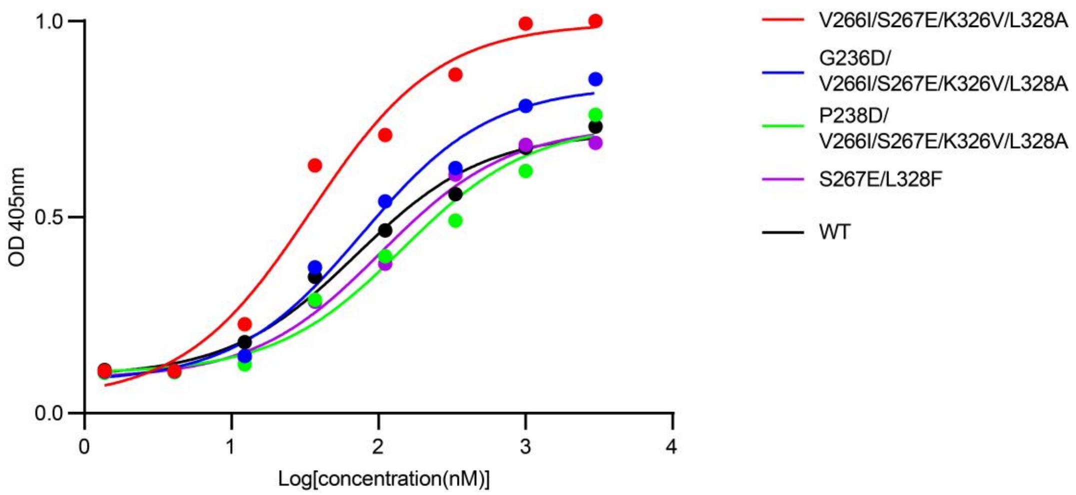

Figure S13. Binding of the human complement component C1q to NK003 variants by ELISA.

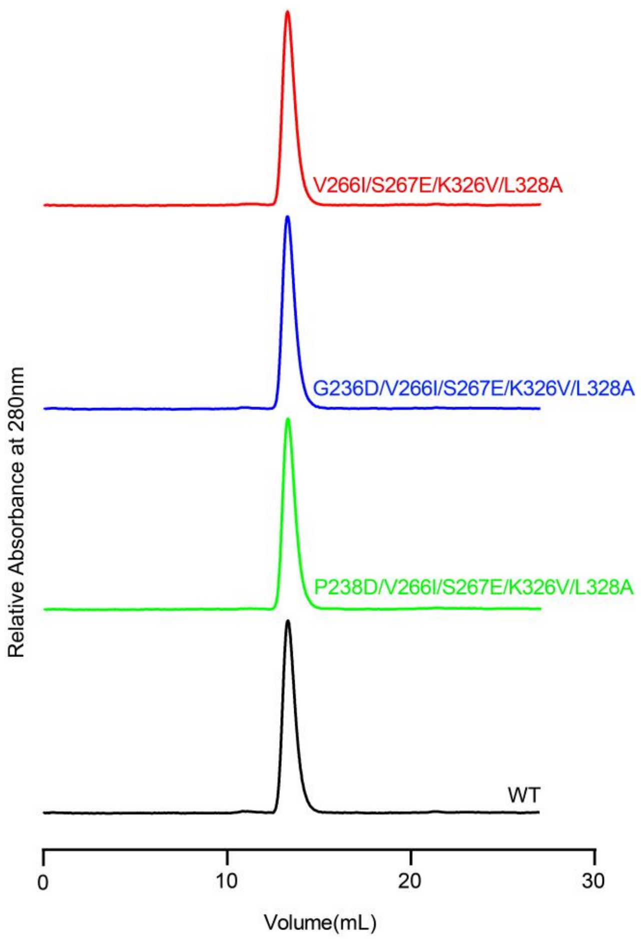

Figure S14. Analysis of NK003 variants by size exclusion chromatography.

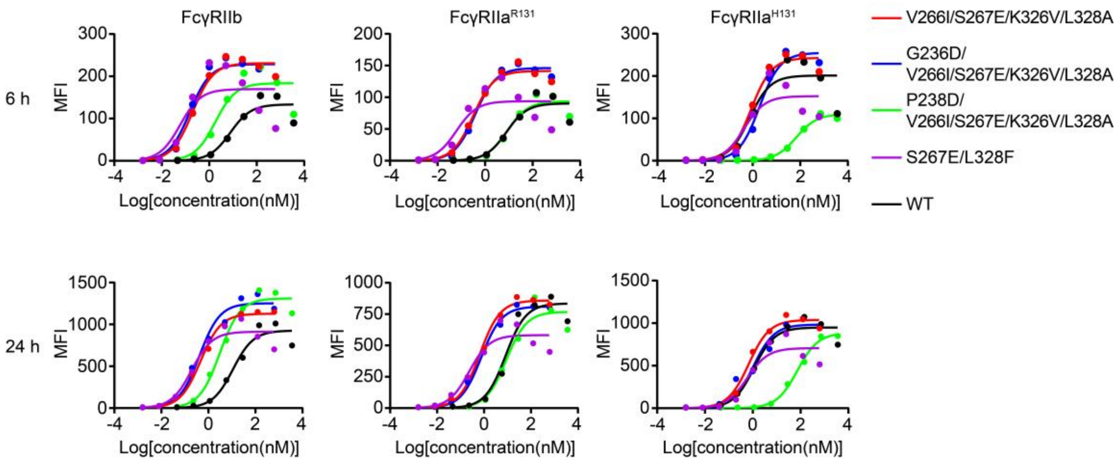

Figure S15. Engineered CD40 agonist antibodies displayed divergent FcγRIIa and FcγRIIb-dependent agonistic activity. CD40 reporter cell analysis of CD40 agonist antibody variants after 6 h (upper row) or 24 h (lower row) as in Figure 6A.

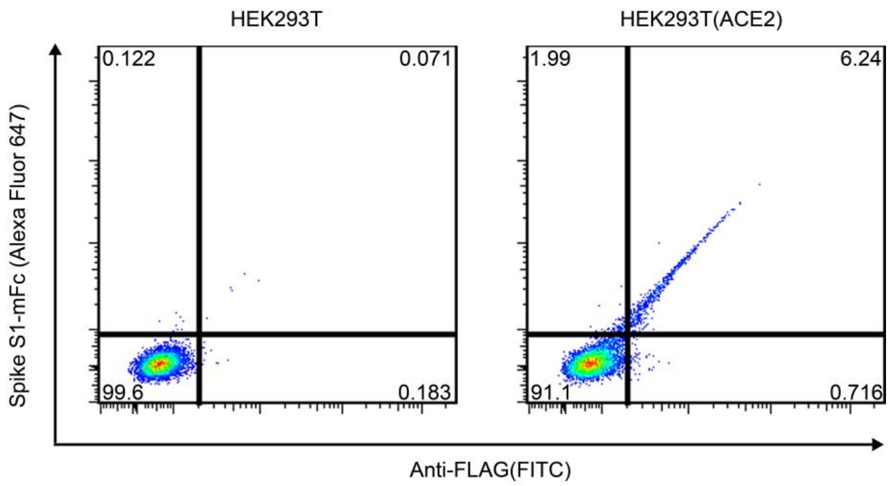

Figure S16. ACE2 displayed on HEK293T cells. HEK293T (left) and HEK293T displaying ACE2 (right) were stained with FITC conjugated anti-FLAG antibody, SARS-CoV-2 Spike S1-mFc protein and rabbit anti-mouse IgG Fc antibody (Alexa Fluor 647). The cells were analyzed by flow cytometry.
